# Seasonal synchronization of foodborne outbreaks in the United States, 1996–2017

**DOI:** 10.1038/s41598-020-74435-9

**Published:** 2020-10-15

**Authors:** Ryan B. Simpson, Bingjie Zhou, Elena N. Naumova

**Affiliations:** Tufts University Friedman School of Nutrition Science and Policy, Boston, USA

**Keywords:** Infectious diseases, Bacterial infection, Risk factors, Scientific data, Statistics, Epidemiology, Population screening, Disease prevention, Public health

## Abstract

Modern food systems represent complex dynamic networks vulnerable to foodborne infectious outbreaks difficult to track and control. Seasonal co-occurrences (alignment of seasonal peaks) and synchronization (similarity of seasonal patterns) of infections are noted, yet rarely explored due to their complexity and methodological limitations. We proposed a systematic approach to evaluate the co-occurrence of seasonal peaks using a combination of L-moments, seasonality characteristics such as the timing (phase) and intensity (amplitude) of peaks, and three metrics of serial, phase-phase, and phase-amplitude synchronization. We used public records on counts of nine foodborne infections abstracted from CDC’s FoodNet Fast online platform for the US and ten representative states from 1996 to 2017 (264 months). Based on annualized and trend-adjusted Negative Binomial Harmonic Regression (NBHR) models augmented with the δ-method, we determined that seasonal peaks of *Campylobacter*, *Salmonella*, and Shiga toxin-producing *Escherichia Coli* (STEC) were tightly clustered in late-July at the national and state levels. Phase-phase synchronization was observed between *Cryptosporidium* and *Shigella*, *Listeria*, and *Salmonella* (ρ = 0.51, 0.51, 0.46; p < 0.04). Later peak timing of STEC was associated with greater amplitude nationally (ρ = 0.50, p = 0.02) indicating phase-amplitude synchronization. Understanding of disease seasonal synchronization is essential for developing reliable outbreak forecasts and informing stakeholders on mitigation and preventive measures.

## Introduction

Globalization of the food supply adds to the challenge of tracking sources of food contamination. According to the Centers for Disease Control and Prevention (CDC), 1 in 6 Americans (or 48 million) become sick from a foodborne infection annually with 3000 deaths^1^. These illnesses often span multiple states, cause extensive revenue losses for food distributors, and result in millions of pounds of recalled foods^2–7^. The US Foodborne Disease Active Surveillance Network (FoodNet), a collaboration among the CDC, state health departments, U.S. Department of Agriculture's Food Safety and Inspection Service (USDA–FSIS), and the Food and Drug Administration (FDA), monitors the nine most prominent foodborne and waterborne illnesses accounting for over 90% of the 9.4 million cases occurring annually^8^. Established in 1996, this surveillance system maintains records for *Campylobacter, Listeria, Salmonella,* Shiga toxin-producing *E. coli (*STEC*), Shigella, Vibrio*, and *Yersinia enterocolitica*. *Cryptosporidium* and *Cyclospora* were added in 1997; and in 2000, STEC non-O157 was added to the list of pathogens commonly transmitted through food^8^.

In the United States, seasonal increases in cases for *Campylobacter*, STEC, *Listeria*, and *Salmonella* during summer months have been demonstrated using FoodNet records^9–11^. The consistency of foodborne infection seasonality with co-occurring seasonal peaks has inspired the creation of “infection calendars”^12–16^. Better understanding of infection seasonality allows for the identification of environmental and manmade drivers of seasonal infection to improve control measures^15,17–19^. Standardized methods for characterizing seasonality features are needed for effective tracking of how seasonal profiles change over time and across large geographic zones^20–22^. These methods can demonstrate if and how nation-wide policy implementation might delay the onset or dampen the intensity of seasonal outbreaks^12^. Furthermore, comparing seasonality features among infections with a common route of transmission may show whether infections with similar seasonal characteristics (peak timing, amplitude, duration) might proxy for one another^13,15,16^ to facilitate the development of new tools for disease forecasting.

The synchronization of seasonal peaks across diseases in the same location or across locations for the same disease is a characteristic of a spatiotemporal pattern of infections with a common route of transmission. The seasonal peaks are merely temporal cumulative clustering of local foodborne outbreaks. In complex system sciences, synchronization is defined as a system’s property in which the dynamics of individual elements of a system are correlated in time due to nonlinear interactions between elements. Many research fields provide illustrations of synchronization, including biology^23^, trade and finance^24^, mathematics^25^, and social sciences^26^. We argue that the synchronization of diseases could be established due to the spatiotemporal alignment of conditions favoring the spread of infections sharing common seasonality features. By extension, infections sharing synchronized seasonal peaks may share similar environmental and manmade drivers of infection associated with food contamination during production and distribution stages.

The synchronization across diseases in a population can be detected and measured. Surveillance systems offer a platform to present time-referenced reported records as an ongoing stream of time series and to assess the properties of these time series, namely the seasonal peak timing and amplitude of seasonal oscillations. The time series of diseases in specific geographic locations can be correlated across infections in a single location or for a single disease across multiple locations. In our earlier works, we illustrated seasonal synchronization between six reported enteric infections in Massachusetts^16^ using a systematic approach for estimating the peak timing and amplitude along with their measures of uncertainty^21,27^ applied to state surveillance records. We also demonstrated a possible relationship between annual peaks and amplitudes: early peaks in influenza are likely to pair with higher amplitude^22^. These models parallel more recent efforts taken by the CDC and FoodNet surveillance reporting teams^28^.

**Figure 1:**
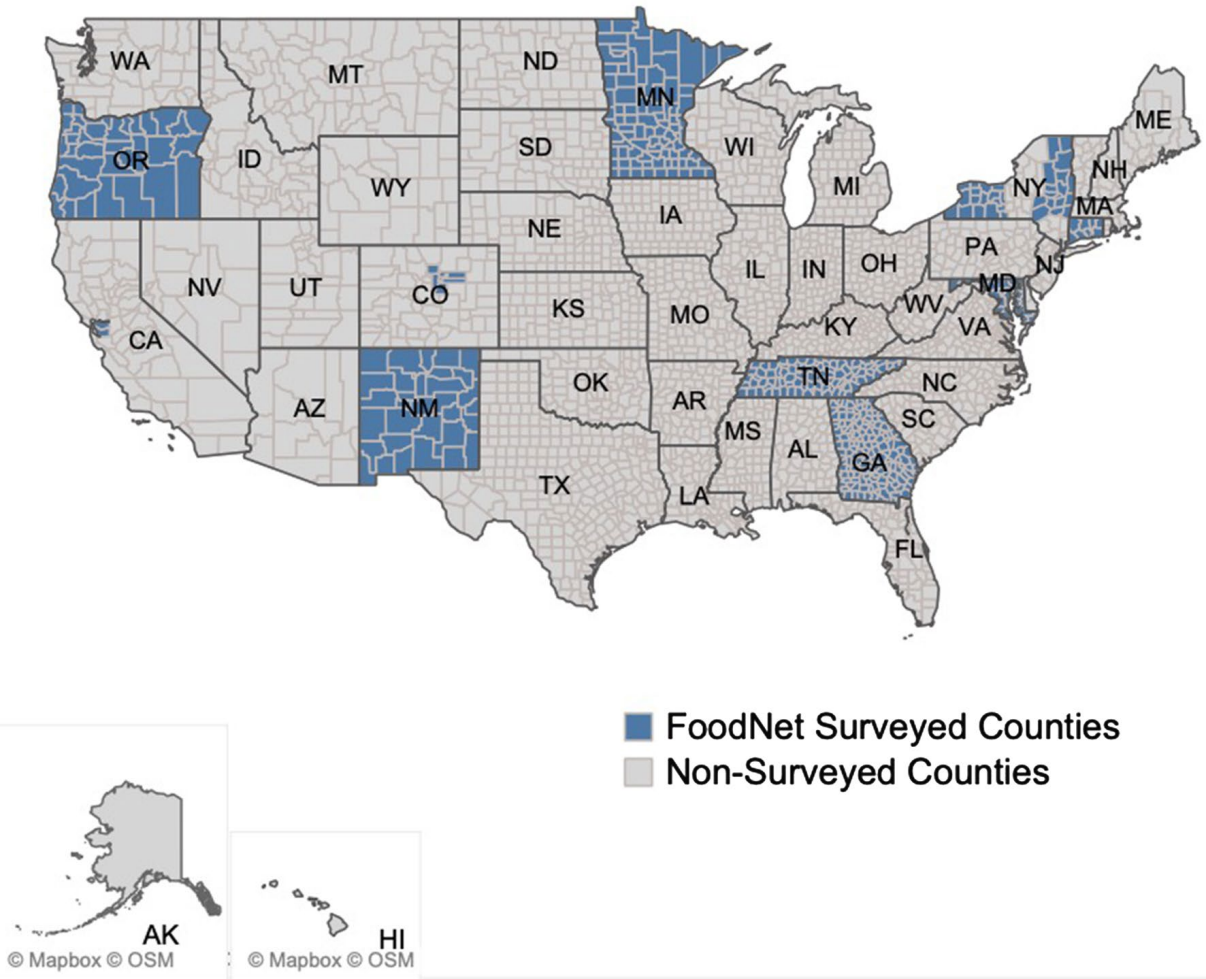
A map of surveyed FoodNet counties as of 2018 (see “Data and methods” section for detail).

In this study, we further developed techniques for defining, characterizing, and comparing seasonal disease outbreaks. We applied the proposed methodology to monthly rates of nine infections reported by FoodNet public sites in the United States and ten surveyed states (as shown in Fig. 1) and all available years (Supplementary Table S1). First, we demonstrated the use of high order characteristics applied to the distribution of monthly rates, specifically L-skewness and L-kurtosis, to identify infections with periodic and non-periodic outbreaks. Next, we developed annualized and trend-adjusted Negative Binomial Harmonic Regression (NBHR) models to estimate the seasonal characteristics for each infection. We applied the δ-methods to derive peak timing and amplitude estimates and their confidence intervals for each infection. We introduced three metrics of outbreak synchronization: serial synchronization based on serial cross-correlations between time series, phase-phase synchronization based on correlations between peak timing estimates, and phase-amplitude synchronization based on correlations across peak timing and amplitude estimates. These techniques allowed us to characterize seasonal infections in a standardized manner, identify possible multi-state outbreaks, and potentially enhance near-time ensemble forecasting. The proposed standardization of data review and analysis is essential for developing reliable outbreak forecasts and informing stakeholders on mitigation and prevention measures, and scheduling food contamination inspections.

## Results

### Monthly rates and sporadic outbreaks

**Figure 2:**
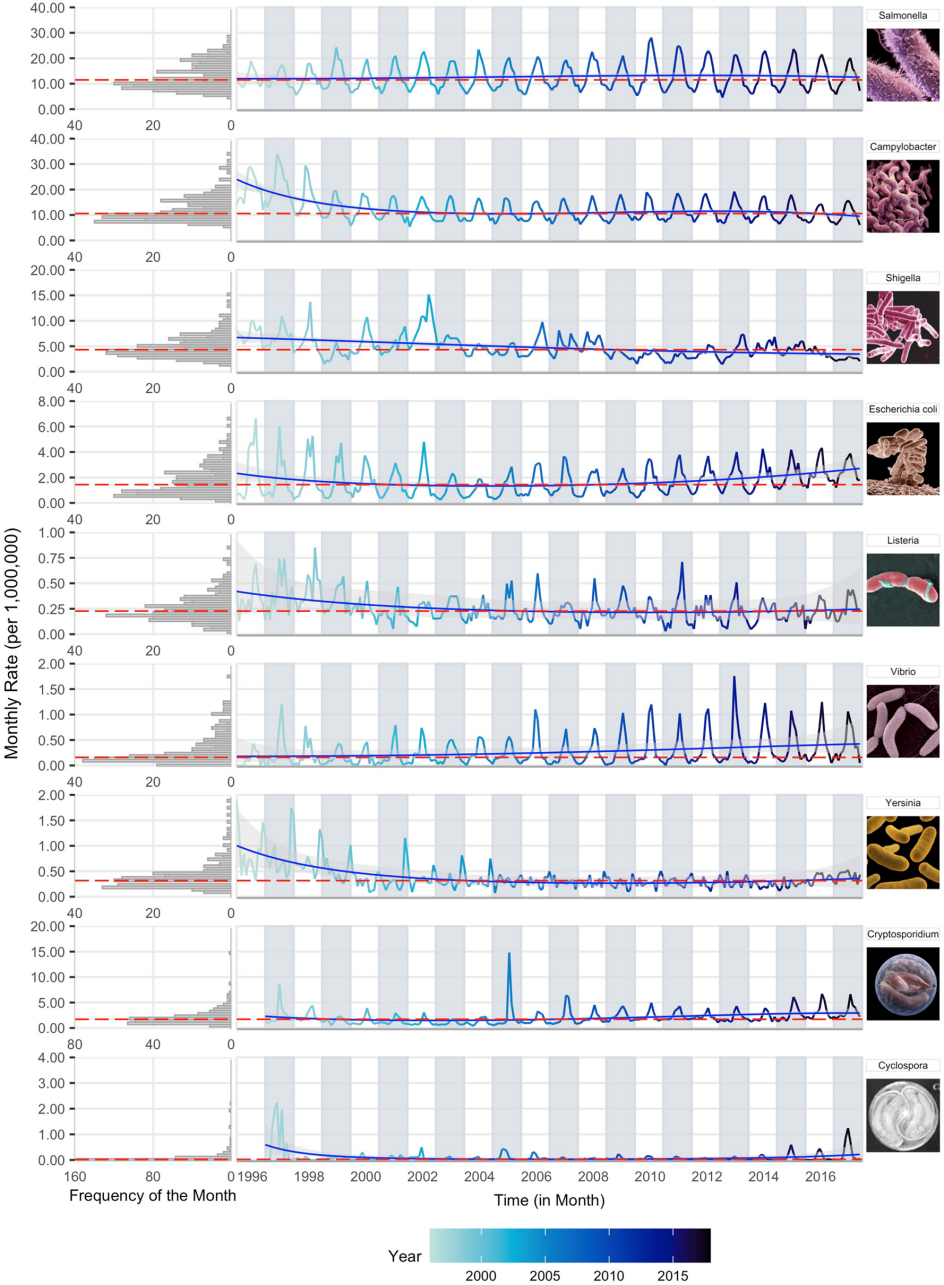
Multi-panel stacked time series plots of monthly reported rates per 1,000,000 persons with median rates (red lines) and predicted trend (blue lines) based on Model 1 and Model 5, respectively, accompanied by the left-rotated frequency histograms for nine FoodNet-reported infections in the United States for available years in 1996–2017. Time series line colour shade indicates more historic vs. more recent data.

The monthly time series of reported rates for nine foodborne infections (seven bacterial and two protozoal) are shown as a multi-panel plot of stacked time series and rotated frequency histograms for the US from 1996 to 2017 (Fig. 2). The right panel provides a time series for visual inspection of the potential periodic nature of the data. The rotated frequency histogram indicates the right-skewness of the monthly rate distribution, justifying the use of negative binomial regression models. The general summary statistics, including GLM-based average monthly rates and the L-skewness and L-kurtosis coefficients, are provided in Table 1. GLM-based monthly rates properly calculate confidence interval estimates and demonstrate differences in intensity across infections and locations. Coefficients of L-skewness and L-kurtosis identify time series distributions with stable seasonal behaviors or sporadic outbreaks typically reflected by low or high values, respectively.

**Table 1:** GLM-based monthly average rates per 1,000,000 persons accompanied by 95% confidence intervals, L-skewness, and L-kurtosis estimates for the nine FoodNet reported infections for the United States and ten surveyed states from 1996 to 2017.

	US	CA	CO	CT	GA	MD	MN	NM	NY	OR	TN
**Bacteria, > 10.0 cpm**											
Salmonella											
LCI	11.18	11.67	8.438	10.22	15.70	11.23	9.976	11.79	8.605	7.537	10.54
Rate	12.68	13.23	9.746	11.59	17.78	12.81	11.31	13.79	9.827	8.562	12.11
UCI	14.37	14.99	11.26	13.14	20.12	14.61	12.83	16.13	11.22	9.726	13.91
L-Skw	0.178	0.197	0.213	0.226	0.228	0.205	0.231	0.256	0.241	0.179	0.193
L-Krt	0.046	0.151	0.163	0.117	0.054	0.097	0.131	0.152	0.144	0.152	0.087
Campylobacter											
LCI	10.74	23.52	10.39	12.66	5.667	6.353	13.57	12.48	10.17	14.20	4.932
Rate	12.17	26.60	11.99	14.34	6.452	7.270	15.37	14.59	11.60	16.08	5.699
UCI	13.80	30.07	13.83	16.25	7.345	8.321	17.40	17.06	13.24	18.21	6.586
L-Skw	0.269	0.175	0.219	0.276	0.264	0.178	0.232	0.181	0.218	0.175	0.147
L-Krt	0.134	0.192	0.153	0.180	0.178	0.108	0.101	0.090	0.115	0.127	0.105
**Bacteria, < 5.0 cpm**											
Shigella											
LCI	4.235	6.642	2.728	1.489	7.654	2.826	3.470	3.941	1.485	1.799	4.609
Rate	4.835	7.551	3.193	1.733	8.693	3.266	3.972	4.655	1.741	2.083	5.330
UCI	5.520	8.586	3.737	2.016	9.874	3.774	4.545	5.500	2.040	2.413	6.164
L-Skw	0.230	0.314	0.373	0.367	0.302	0.514	0.511	0.297	0.444	0.337	0.384
L-Krt	0.153	0.186	0.251	0.280	0.195	0.395	0.331	0.209	0.313	0.210	0.167
STEC											
LCI	1.470	1.381	2.086	1.428	0.540	0.674	3.155	1.355	1.455	2.243	1.050
Rate	1.711	1.610	2.455	1.663	0.654	0.814	3.616	1.641	1.707	2.585	1.256
UCI	1.992	1.878	2.889	1.938	0.792	0.983	4.144	1.988	2.002	2.980	1.502
L-Skw	0.242	0.330	0.282	0.323	0.234	0.196	0.277	0.215	0.371	0.299	0.254
L-Krt	0.098	0.198	0.161	0.225	0.141	0.119	0.099	0.141	0.267	0.155	0.142
**Bacteria, < 0.5 cpm**											
Listeria											
LCI	0.196	0.297	0.154	0.376	0.152	0.217	0.098	0.137	0.257	0.189	0.106
Rate	0.256	0.374	0.213	0.466	0.204	0.284	0.138	0.198	0.331	0.248	0.153
UCI	0.334	0.472	0.296	0.577	0.273	0.372	0.196	0.287	0.427	0.325	0.221
L-Skw	0.206	0.255	0.570	0.299	0.229	0.245	0.458	0.640	0.377	0.282	0.268
L-Krt	0.160	0.146	0.276	0.135	0.124	0.108	0.132	0.335	0.179	0.108	0.042
Vibrio											
LCI	0.208	0.515	0.134	0.331	0.166	0.389	0.110	0.044	0.123	0.221	0.085
Rate	0.270	0.625	0.189	0.414	0.221	0.486	0.153	0.078	0.174	0.286	0.126
UCI	0.351	0.759	0.267	0.518	0.293	0.606	0.213	0.140	0.246	0.369	0.188
L-Skw	0.394	0.510	0.540	0.548	0.265	0.399	0.563	0.694	0.539	0.609	0.417
L-Krt	0.198	0.247	0.192	0.280	0.124	0.123	0.265	0.349	0.209	0.306	0.110
Yersinia											
LCI	0.305	0.415	0.173	0.314	0.396	0.147	0.327	0.067	0.275	0.337	0.206
Rate	0.384	0.510	0.237	0.394	0.489	0.200	0.409	0.110	0.352	0.420	0.274
UCI	0.482	0.628	0.324	0.494	0.604	0.272	0.512	0.181	0.451	0.525	0.366
L-Skw	0.366	0.342	0.395	0.274	0.529	0.300	0.258	0.685	0.235	0.207	0.278
L-Krt	0.291	0.195	0.061	0.111	0.407	0.069	0.178	0.352	0.075	0.084	0.170
**Protozoa, < 5.0 cpm**											
Cryptosporidium											
LCI	1.758	1.564	1.076	0.956	1.850	0.580	3.740	3.118	1.825	2.202	0.974
Rate	2.044	1.824	1.292	1.133	2.164	0.706	4.290	3.697	2.127	2.547	1.168
UCI	2.376	2.127	1.551	1.342	2.532	0.859	4.920	4.384	2.480	2.947	1.401
L-Skw	0.334	0.436	0.385	0.353	0.210	0.322	0.345	0.356	0.619	0.280	0.415
L-Krt	0.236	0.270	0.265	0.225	0.183	0.229	0.210	0.237	0.551	0.100	0.306
**Protozoa, < 0.5 cpm**											
Cyclospora											
LCI	0.065	0.085	0.071	0.241	0.151	0.039	0.037	0.039	0.026	0.013	0.011
Rate	0.098	0.141	0.133	0.311	0.206	0.068	0.074	0.078	0.055	0.036	0.028
UCI	0.148	0.235	0.249	0.400	0.279	0.119	0.146	0.157	0.116	0.106	0.070
L-Skw	0.697	0.853	0.853	0.740	0.724	0.803	0.805	0.802	0.873	0.865	0.810
L-Krt	0.508	0.682	0.680	0.501	0.481	0.587	0.581	0.561	0.715	0.690	0.578

*Note* LCI and UCI are lower and upper boundaries for the 95% confidence interval (CI), respectively; L-Skw represents L-skewness while L-Krt represents L-kurtosis estimates.

Nationally, the monthly rates of infections exhibited marked variability of 100-fold difference from ~ 20 to ~ 0.2 cases per month per 1,000,000 persons (cpm). Based on the average monthly rates and their variability we clustered infections in three distinct groups. *Salmonella* and *Campylobacter* infections had the highest average monthly rates of above 10.0 cpm: 12.68 [11.18, 14.37] and 12.17 [10.74, 13.80] cpm, respectively. *Shigella, Cryptosporidium,* and STEC had average monthly rates under 5.0 cpm: 4.84 [4.24, 5.52], 2.04 [1.76, 2.38], and 1.71 [1.47, 1.99] cpm, respectively. *Yersinia, Vibrio*, *Listeria,* and *Cyclospora* had the lowest average monthly rates under 0.5 cpm.

The reported monthly rates of infections vary substantially across states at the magnitude of two to eightfold. For infections with overall high rates the fold increase across states was under 5-folds*.* For example, *Campylobacter* shows ~ 4.6-fold change with highest average monthly rates in CA and lowest in TN (26.6 [23.5, 30.1] cpm vs 5.70 [4.93, 6.59] cpm). For *Shigella* there was ~ 4.4-fold change with highest rates in CA and lowest in CT (7.55 [6.64, 8.59] vs 1.73 [1.49, 2.02] cpm); *Salmonella* shows ~ twofold change with rates ranging from 17.8 [15.7, 20.1] cpm in GA to 8.56 [7.54, 9.73] cpm in OR. For protozoal infections, *Cryptosporidium* had the highest rates in MN (4.29 [3.74, 4.92] cpm), and lowest rates in MD (0.71 [0.58, 0.86] cpm), yielding sixfold change. *Cyclospora* exhibited the fold increase of 7.4.

As expected, the occurrence of sporadic outbreaks of high intensity was most notable at the state level. The stability of seasonal outbreaks was well detected by L-skewness and L-kurtosis coefficients: low coefficients were found for infections with overall high rates and stable seasonality while high coefficients were found for infections with low monthly rates and sharp sporadic outbreaks. Nationally for *Salmonella*, these coefficients were 0.18 and 0.05, respectively, demonstrating a stable, seasonal pattern. In NM, NY and MN, L-skewness exceeded the national estimate by ~ 1.3 times and in CO, NM, and OR L-kurtosis exceeded the national estimate by ~ 3.0 times, indicating the presence of spikes with increased intensity. For infections with overall low rates, like *Yersinia, Vibrio*, *Cryptosporidium,* and *Cyclospora*, the large values of L-skewness and L-kurtosis coefficients indicate a frequent occurrence of irregular spikes.

### Seasonality analysis

As shown in Fig. 2 and supported by L-moments in Table 1, most of the infections exhibited regular periodic increases in incidence indicative of seasonality. Therefore, we estimated peak timing and amplitude based on Model 2 and results are shown in Table 2 (see Supplementary Table S3 for details). Nationwide, all infections except *Yersinia* exhibited summer peaks ranging from early-June (6.43-month for *Cyclospora*) to mid-August (8.59-month for *Shigella*). Four infections: *Campylobacter, Vibrio, Salmonella*, and STEC peaked during mid- to late-July (7.30 [7.08, 7.53]; 7.72 [7.59, 7.84]; 7.79 [7.71, 7.87]; and 7.81 [7.66, 7.96], respectively). The peak in *Campylobacter* was significantly earlier than *Vibrio* (p = 0.015), *Salmonella* (p < 0.001), and STEC (p = 0.005). Three infections: *Cryptosporidium, Listeria*, and *Shigella* peaked during August (8.19 [7.96, 8.43]; 8.35 [8.05, 8.65]; and 8.59 [8.07, 9.11], respectively). Peak timing for *Yersinia* was inconclusive; peak timing of *Cyclospora* was highly variable (6.43 [5.86, 7.01]). Nationally, the amplitude of seasonal peaks varies from 1.27 [1.12, 1.43] for *Yersinia* to 5.56 [3.55, 8.72] for *Cyclospora.*

**Table 2:** Average peak timing in months and amplitude and their 95% confidence intervals for monthly rates per 1,000,000 persons for nine infections reported by FoodNet in the United States and ten surveyed states from 1996 to 2017.

	US	CA	CO	CT	GA	MD	MN	NM	NY	OR	TN
**Bacteria, > 10.0 cpm**											
*S*almonella											
LCI	7.713	7.339	7.175	7.198	8.124	7.513	7.061	7.536	7.050	6.777	7.700
PT	7.793	7.558	7.528	7.430	8.236	7.649	7.260	7.845	7.226	7.078	7.841
UCI	7.874	7.777	7.881	7.663	8.349	7.784	7.458	8.153	7.402	7.379	7.982
LCI	1.678	1.425	1.317	1.559	2.031	1.712	1.470	1.666	1.710	1.318	1.768
AMP	1.704	1.470	1.377	1.615	2.079	1.757	1.515	1.730	1.776	1.373	1.818
UCI	1.731	1.514	1.438	1.672	2.127	1.802	1.560	1.794	1.841	1.428	1.868
Campylobacter											
LCI	7.077	6.887	7.051	6.989	6.669	7.023	7.253	7.571	7.297	6.986	6.983
PT	7.304	7.528	7.291	7.224	6.973	7.272	7.408	7.809	7.491	7.188	7.196
UCI	7.530	8.168	7.530	7.459	7.277	7.521	7.563	8.048	7.686	7.390	7.410
LCI	1.454	1.152	1.523	1.520	1.401	1.551	1.651	1.734	1.579	1.410	1.476
AMP	1.502	1.211	1.591	1.572	1.463	1.615	1.689	1.794	1.630	1.454	1.525
UCI	1.550	1.270	1.659	1.624	1.524	1.678	1.726	1.854	1.681	1.498	1.575
**Bacteria, < 5.0 cpm**											
Shigella											
LCI	8.070	7.788	8.450	6.631	7.782	6.550	6.696	8.401	7.308	8.315	7.440
PT	8.589	8.558	8.834	7.629	8.812	7.611	7.824	8.949	8.657	9.221	9.360
UCI	9.108	9.328	9.219	8.627	9.842	8.672	8.953	9.498	10.01	10.13	11.28
LCI	1.236	1.208	1.779	1.141	1.148	1.306	1.156	1.497	1.149	1.219	0.991
AMP	1.305	1.317	1.931	1.296	1.250	1.510	1.369	1.636	1.355	1.355	1.154
UCI	1.374	1.426	2.083	1.451	1.351	1.714	1.582	1.775	1.562	1.491	1.318
STEC											
LCI	7.655	7.800	7.074	6.977	6.817	7.626	7.661	7.262	7.604	7.864	6.916
PT	7.806	8.262	7.429	7.411	7.232	7.942	7.836	7.611	7.995	8.083	7.299
UCI	7.957	8.724	7.784	7.845	7.647	8.257	8.011	7.960	8.385	8.301	7.683
LCI	2.233	1.732	1.802	1.758	1.743	1.939	2.613	1.864	2.198	2.406	1.747
AMP	2.306	1.904	1.968	1.932	1.875	2.066	2.714	2.000	2.396	2.542	1.873
UCI	2.379	2.076	2.133	2.106	2.008	2.194	2.815	2.136	2.594	2.679	2.000
**Bacteria, < 0.5 cpm**											
Listeria											
LCI	8.048	4.292	8.025	7.794	5.229	7.800	8.736	7.895	8.028	7.755	7.019
PT	8.351	7.027	8.648	8.196	6.481	8.345	9.383	8.755	8.627	8.317	8.624
UCI	8.654	9.762	9.272	8.597	7.733	8.889	10.03	9.614	9.226	8.879	10.23
LCI	1.497	0.937	1.949	1.832	1.092	1.571	1.752	2.034	1.854	1.611	1.052
AMP	1.572	1.118	2.406	2.018	1.257	1.742	2.012	2.596	2.079	1.793	1.263
UCI	1.646	1.298	2.863	2.203	1.423	1.913	2.273	3.158	2.305	1.974	1.475
Vibrio											
LCI	7.592	7.707	7.328	7.560	7.257	7.337	7.178	7.388	7.265	7.652	6.977
PT	7.718	7.914	7.764	7.786	7.667	7.499	7.628	8.648	7.603	7.844	7.459
UCI	7.844	8.121	8.200	8.011	8.076	7.662	8.078	9.907	7.941	8.036	7.940
LCI	3.893	5.540	3.182	4.867	2.113	5.644	2.417	1.697	3.611	8.254	2.227
AMP	4.020	5.817	3.576	5.211	2.269	5.851	2.742	2.258	3.999	8.743	2.508
UCI	4.148	6.095	3.969	5.554	2.426	6.059	3.066	2.818	4.387	9.232	2.789
Yersinia											
LCI	12.34	8.203	5.989	3.295	12.47	12.62	5.906	6.790	1.615	1.835	12.75
PT	1.089	12.51	7.755	7.481	12.84	2.612	7.204	8.648	4.233	3.380	1.461
UCI	1.841	5.814	9.521	11.67	1.222	5.608	8.502	10.51	6.850	4.924	2.173
LCI	1.144	0.862	1.080	0.905	2.185	0.953	1.109	1.000	0.960	1.033	1.460
AMP	1.265	1.069	1.337	1.085	2.433	1.144	1.246	1.558	1.135	1.196	1.654
UCI	1.386	1.277	1.595	1.265	2.681	1.336	1.383	2.117	1.310	1.359	1.847
**Protozoa, < 5.0 cpm**											
Cryptosporidium											
LCI	7.959	6.110	7.801	7.882	8.004	8.012	7.726	7.625	7.997	7.166	8.001
PT	8.194	8.635	8.256	8.251	8.404	8.531	7.974	8.063	8.318	8.094	8.478
UCI	8.430	11.16	8.711	8.619	8.804	9.051	8.223	8.501	8.638	9.023	8.956
LCI	1.724	0.959	1.757	1.935	1.336	1.526	2.013	1.715	2.728	1.206	1.684
AMP	1.833	1.144	1.991	2.107	1.429	1.698	2.121	1.915	3.166	1.352	1.890
UCI	1.942	1.330	2.226	2.278	1.521	1.871	2.229	2.115	3.604	1.498	2.097
**Protozoa, < 0.5 cpm**											
Cyclospora											
LCI	5.857	5.402	6.142	6.096	6.239	6.422	6.279	5.381	6.247	3.657	5.889
PT	6.432	5.957	6.611	6.386	7.432	7.095	6.792	6.057	6.739	7.145	6.537
UCI	7.007	6.511	7.081	6.676	8.625	7.769	7.306	6.732	7.230	10.63	7.184
LCI	5.114	4.979	37.78	9.872	1.705	4.057	9.172	6.426	9.850	0.578	8.577
AMP	5.563	6.151	40.65	10.45	2.187	4.838	10.35	7.870	11.61	1.651	9.516
UCI	6.012	7.323	43.51	11.12	2.668	5.618	11.54	9.313	13.38	2.723	10.46

*Note* LCI and UCI are lower and upper boundaries for the 95% confidence interval (CI), respectively, for peak timing (PT) and amplitude (AMP) estimates.

We compared peak timing estimates to identify seasonal co-occurrences and determine the potential for phase-phase synchronization. At the state level, *Salmonella* peaked during July for all states except GA (8.24 [8.12, 8.35]) while *Campylobacter* had the earliest peak in GA and latest in NM (Table 2). The peak of *Campylobacter* in GA significantly (p < 0.03) preceded peaks in six states: CA, CO, MN, NM, NY, and OR. Similarly, the peak of *Campylobacter* in CA significantly (p < 0.022) succeeded peaks in seven states: CO, CT, MD, MN, NY, OR, and TN. *Listeria* had far more state-level variability between mid-June in GA to mid-September in MN. STEC also had large state-level variability (early-July in GA to mid-August in CA). *Cryptosporidium* had the least variability across states (from MN (7.97 [7.73, 8.22]) to CA (8.64 [6.11, 11.16]), yet its peak timing in CT significantly succeeded peaks in MN (p = 0.025) and OR (p = 0.048). Although nationally *Yersinia* peaked at the beginning of January, peaks were spread sporadically across states. Like *Yersinia*, state-specific seasonal peaks for *Cyclospora* were spread over 2 months*.*

Across states, *Salmonella, Campylobacter, Shigella,* and STEC had the least variability of amplitude estimates, with about 30–70% difference between the highest and lowest amplitudes (Table 2)*.* The high values of peak amplitude for *Salmonella* in GA and for *Campylobacter* in NM tended to co-occur with late peak timing. *Cyclospora* and *Vibrio* had the largest amplitude variability. The large values of peak amplitude co-occur with high values for skewness and kurtosis for *Vibrio* in NY and for *Cyclospora* in OR.

**Figure 3:**
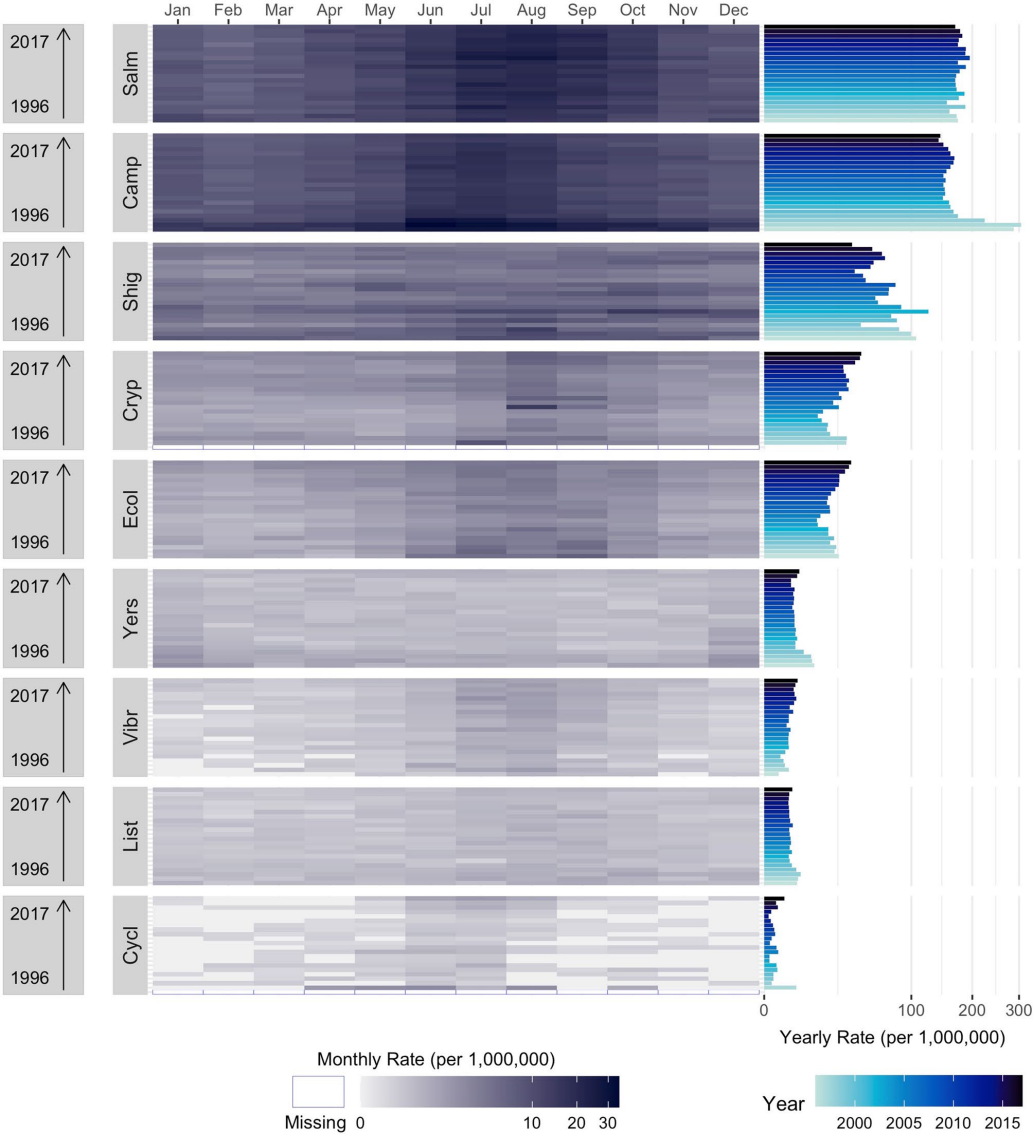
Multi-panel calendar plots of monthly rates combined with yearly rates and peak timing (in month) and amplitude average estimates for nine infections: *Salmonella* (Salm), *Campylobacter* (Camp), *Shigella* (Shig), Shiga Toxin-producing *E. coli* (Ecol), *Listeria* (List), *Vibrio* (Vibr), *Yersinia* (Yers), *Cryptosporidium* (Cryp), and *Cyclospora* (Cycl) as reported by FoodNet in the United States from 1996 to 2017. Average peak timing and amplitude estimates are shown in the top panel, annual trends in rates are shown in the right-rotated bar-charts, and heat maps indicate monthly rates for each infection in the main panel.

To depict the relationship between peak timing and amplitude simultaneously with annual trend and reoccurrences of seasonal changes for all infections in the US, we combined the traditional time series as a multi-panel calendar plot (Fig. 3). Based on the relationship between peak timing and amplitude, all infections exhibited summer peaks in tightly formed clusters except *Vibrio* and *Yersinia*. The heat map of monthly rates for *Salmonella*, *Campylobacter*, *Shigella,* STEC, and *Cryptosporidium* exhibited distinct seasonal changes in summertime rates.

### Trend analysis

The results of trend analyses are shown in Fig. 2 and Supplementary Tables S4–S6. In Fig. 2, the medians and the predicted trends along with their confidence intervals were obtained from Model 1 and Model 5, respectively. The contribution of trend and seasonal components for each infection is shown in Supplementary Table S4. On the national level, seasonality explains a substantial fraction of variability for six infections: *Salmonella*, STEC, *Vibrio*, *Campylobacter*, *Listeria*, and *Cryptosporidium* (64, 43, 43, 29, 20, and 20%, respectively). The contribution of the trend components was most pronounced in *Campylobacter*, *Yersinia*, *Cyclospora*, and *Vibrio* (48, 29, 25, and 20%, respectively). The contributions of linear, quadratic, and cubic trend components are shown in Supplementary Table S5. Overall, an adjustment for linear and non-linear trend components resulted in < 3% fluctuations in average peak timing and amplitude estimates with < 10% fluctuations for *Cyclospora*, *Yersinia,* and *Cryptosporidium* estimates as shown in Supplementary Table S6. This high stability in seasonality estimates irrespective of trend specifications justified the use of Model 1 for calculating annualized seasonality characteristics and conducting phase-phase and phase-amplitude synchronization analyses.

**Figure 4:**
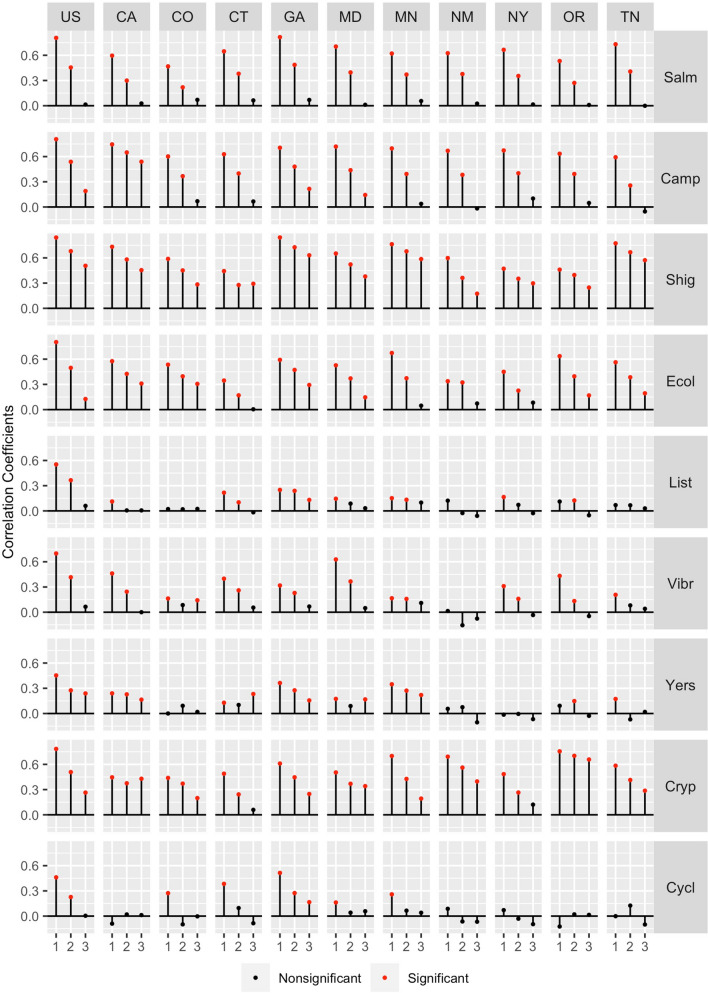
The autocorrelation coefficients across lags of 1–3 months for nine infections: *Salmonella* (Salm), *Campylobacter* (Camp), *Shigella* (Shig), Shiga Toxin-producing *E. coli* (Ecol), *Listeria* (List), *Vibrio* (Vibr), *Yersinia* (Yers), *Cryptosporidium* (Cryp), and *Cyclospora* (Cycl) as reported by FoodNet in the United States and ten surveyed states. Significant values are shown in red.

It is expected that infections with strong trend and/or seasonality components have high autocorrelation, e.g. high dependency on the prior month value, which serves as the base for near-term forecasting. We plotted the correlation coefficients across lags of 1–3 months for each infection in each state and all states combined (Fig. 4; Supplementary Table S7). Nationwide, six infections: *Salmonella, Campylobacter, Shigella,* STEC, *Cryptosporidium* and *Vibrio* had strong autocorrelations at 1-month lag (ρ ≥ 0.70; p < 0.001) and moderate autocorrelations at lag of 2 months (ρ ≥ 0.42; p < 0.001). At the state level, GA had the strongest autocorrelation patterns for *Salmonella* and *Shigella*. CA had the strongest patterns for *Campylobacter*, MN had the strongest patterns for STEC, and OR had the strongest patterns for *Cryptosporidium*. In general, autocorrelations across states for *Listeria* and *Vibrio* were low across most lags (ρ < 0.45) and thus, support low seasonality and trend contributions as shown in Supplementary Table S4.

### Synchronization analysis

**Figure 5:**
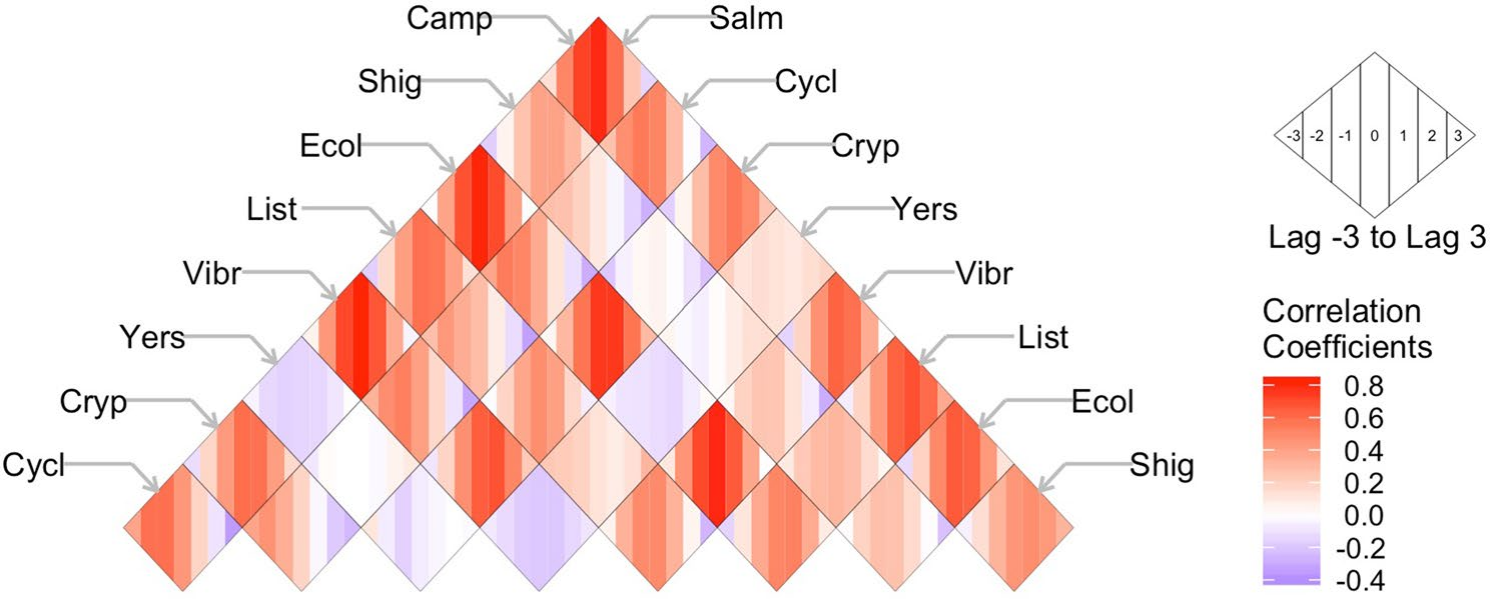
Correlation coefficients across seven lags (− 3 to + 3) between pairs of nine infections *Salmonella* (Salm), *Campylobacter* (Camp), *Shigella* (Shig), Shiga Toxin-producing *E. coli* (Ecol), *Listeria* (List), *Vibrio* (Vibr), *Yersinia* (Yers), *Cryptosporidium* (Cryp), and *Cyclospora* (Cycl) as reported by FoodNet for the US from 1996 to 2017. Infection pairings are found at the intersection of diagonal rows. Shading intensity indicates the strength of correlations ranging from positive (red) to negative (blue) for each lag as a sequence of coloured stripes as shown in the inset.

It is expected that infections with similar trend and seasonality patterns have high cross-correlations, indicating potential synchronization of the shared temporal behavior. Cross-correlation estimates between diseases at − 3 to + 3 months lags are shown in Fig. 5 and Supplementary Table S8. Nationally, monthly rates of *Campylobacter* were strongly correlated with *Salmonella* at lags − 1 to + 2 (ρ = 0.59, 0.78, 0.73, 0.50; p < 0.001). *Campylobacter* was also moderately correlated at lags − 1 and 0 with STEC (ρ = 0.63, 0.66; p < 0.001) and *Vibrio* (ρ = 0.58, 0.63; p < 0.001), and with *Listeria* at lags − 2 to 0 (ρ = 0.60, 0.69, 0.63; p < 0.001). *Salmonella* was also strongly correlated from lags − 1 to + 1 with STEC and *Vibrio* as well as moderately correlated with Listeria at lags − 1 and 0. STEC was strongly correlated with *Vibrio* from lags − 1 to + 1 as well as *Cryptosporidium* from lags − 2 to + 1. *Vibrio* was similarly moderately correlated with *Cryptosporidium* from lags − 2 to 0. These results reaffirm strong similarities in the seasonal patterns across infections.

Nationwide, phase-phase synchronization was most pronounced between *Cryptosporidium* and *Shigella* (ρ = 0.51, p = 0.019), *Listeria* (ρ = 0.51, p = 0.019), and *Salmonella* (ρ = 0.46, p = 0.036) (Supplementary Table S9). Strong positive synchronization between *Cryptosporidium* and *Shigella* indicates concordance in their seasonal behavior; when one peaks later, the other does also (Supplementary Fig. S1). In contrast, no significant synchronization was found between *Salmonella* and *Campylobacter* (ρ = 0.07) (Supplementary Fig. S2), indicating that the seasonal processes of *Salmonella* and *Campylobacter* peak timing are not associated despite peak co-occurrence in July. At the state level, phase-phase synchronization varies and for one pair the correlation could be significant and positive for one state and negative for another, like *Campylobacter-*STEC in CT and GA, indicative of discordant patterns. The strongest correlations were found between *Salmonella* and STEC in NM (ρ = 0.62, p = 0.018) and between *Salmonella* and *Campylobacter* in MN (ρ = 0.60, p = 0.003).

**Figure 6:**
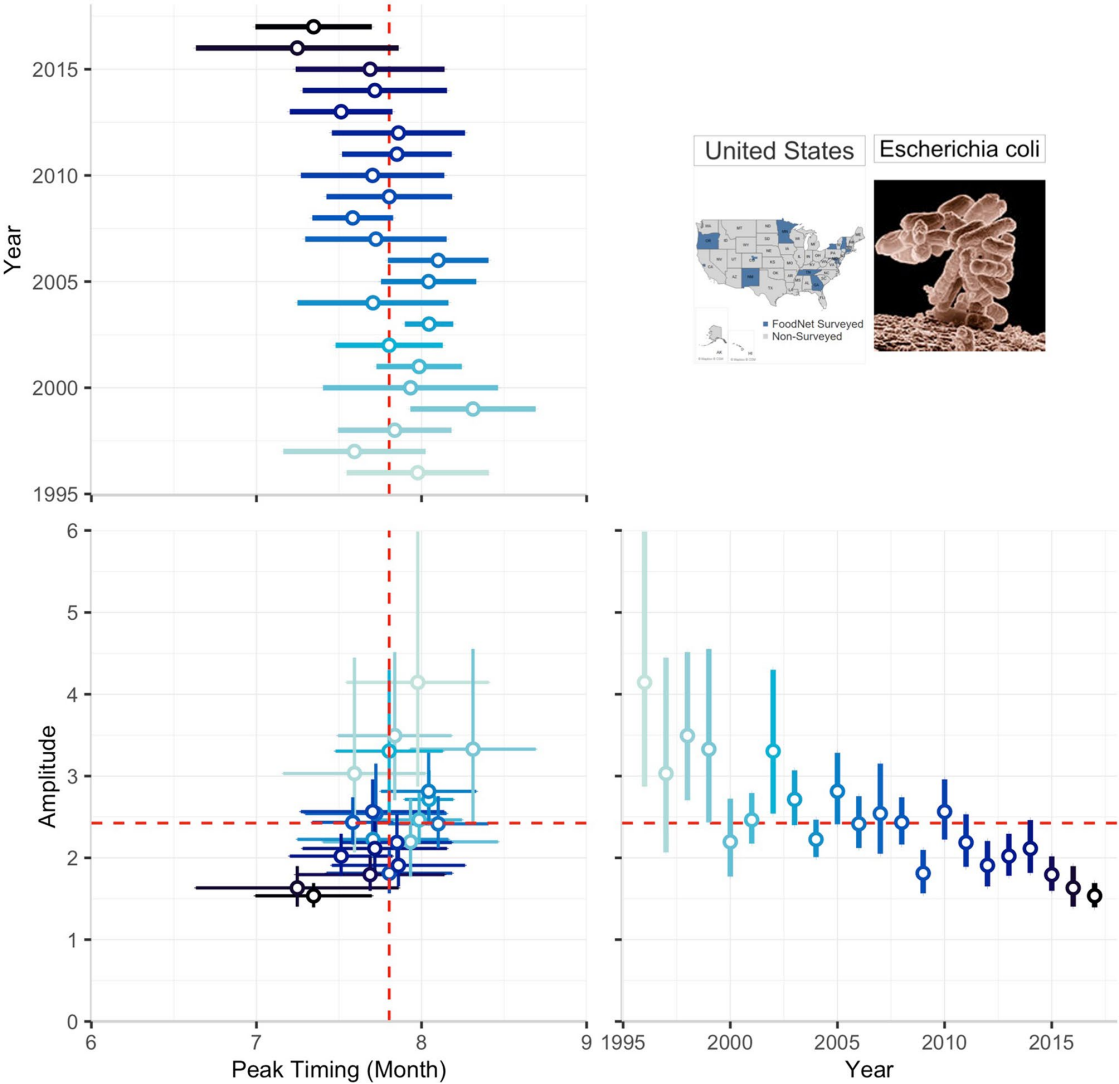
The relationship between annual peak timing and amplitude estimates for Shiga Toxin-producing *E. coli* (STEC) in the US in 1996 to 2017. The multi-panel plot consists of two panels depicting forest plots for peak timing (upper panel) and amplitude (left panel) estimates along with their 95% confidence intervals by year, and the main panel demonstrating the associations between these two seasonality characteristics. Dashed red lines indicate the average peak timing and average amplitude across all years. The colour shade indicates the year of reporting and helps to note the decline in amplitude and the overall tendency to early peak timing that explain the nature of the observed positive correlation and potential synchronization over time.

Examining phase-amplitude synchronization, we found positive correlations indicating that the magnitude of seasonal peaks is likely to increase when an infection peaks later in the year for STEC at the national level (ρ = 0.50, p = 0.019; Fig. 6), as well as in GA (ρ = 0.58, p = 0.005) (Supplementary Table S10). We also found positive correlations for *Shigella* in CA (ρ = 0.63, p = 0.002), GA (ρ = 0.50, p = 0.019), and MN (ρ = 0.43, p = 0.049) as well as for *Cryptosporidium* in CT (ρ = 0.48, p = 0.029) indicating the phase-amplitude synchronization.

## Discussion

Our results demonstrated how rich and powerful tools of time series analyses could be applied to explore the seasonality and synchronization of foodborne infections between one another and across locations. We urge food safety and public health professionals to make efforts to improve and standardize the analysis of reported infections to allow for a meaningful comparison and actionable inferences derived from this analysis^29^. Measures of L-skewness and L-kurtosis, indicating the degree of departure from a well-defined bell-shaped distribution of cases per selected time unit, could be implemented in routine surveillance system data analysis to quantify the overall degree of outbreak intensity and to distinguish between consistent and thus predictable seasonal behaviors and potentially sporadic outbreaks of foodborne infection. Standardized approaches to quantify seasonal peak timing and intensity (amplitude) along with their uncertainty measures agnostic to infection type or geographic location allow for uniform comparison of seasonal patterns common for all or almost all mandatory or voluntary reported infections.

Detection of foodborne infection outbreaks relies on standardized methods for calculating and comparing infection rates and seasonality features that should be implemented with the highest possible precision. The outdated techniques based on arithmetical means produce meaningless negative values for sporadically occurring infections (as shown in Supplementary Table S2) and must be replaced with GLM-calculated estimates based on the highly skewed nature of time series rates. The commonly used aggregation of daily or weekly counts into monthly values is a substandard solution, because it leads to information loss, coarse resolution, and poor understanding of uncertainties needed for proper trend analysis^30^.

By extending the δ-method for systematically estimating the seasonality characteristics, such as peak timing and amplitude, we avoided the traps of using poorly defined seasons, which may vary geographically, climatically, and contextually. When peak timing is calculated as the month with maximal rates using multi-month periods^31–36^, this approach, though computationally straightforward, reduces the precision of estimating seasonal characteristics and neglects temporal and geographic variability^37–43^. Our results show that *Campylobacter*, *Vibrio*, *Salmonella*, and STEC peak from mid- to late-July, suggesting co-occurrence based on month of maximal rate calculations. By applying the δ-method and formal statistical testing we demonstrated that, while all infections peak during July, *Campylobacter* significantly precedes *Vibrio*, *Salmonella*, and STEC. Thus, we are able to identify an important feature, missed by the commonly used methods, yet valuable for disease forecasting.

The standardization of analytical tools could substantially improve our understanding of the co-occurrence of infections with respect to each other and across locations. High values of L-skewness and L-kurtosis spotted an outbreak of *Salmonellosis* in NY in 1996 with 5-times the amplitude than any other year following^44,45^. High L-moment estimates in GA for *Yersinia* indicated erratic outbreaks during December and January months and likely reflect reported outbreaks in pork chitterlings for Christmas and New Year celebrations^46,47^. High skewness and kurtosis values for *Cryptosporidium* and *Cyclospora* aligned with well documented outbreaks in NY and CA^46–51^. Unfortunately, cases reported by the FoodNet Fast platform are designated to a single state, heavily aggregated, and no information was available for multistate outbreaks. FoodNet Fast does not provide granular population catchment information within each county and the counties included in FoodNet represent only a fraction of the total state. Drawing spatial relationships for a single infection risks over-stating the association between states, especially when counties in two states share no geographic border. Improved data collection and reporting will enable modeling and forecasting of foodborne infections using complex network analyses to trace supply chain distribution patterns^52–54^.

This study provides evidence for potential outbreak synchronization based on several metrics that utilized the complex systems thinking. Serial synchronization examines whether two infections share similar trend and seasonality. Phase-phase and phase-amplitude synchronization evaluate shared seasonal processes between two infections peak timing or a single infection’s peak timing and amplitude, respectively. Additionally, these metrics can help provide important information for adapting near-term forecasts to more accurately predict, plan for, and prevent seasonal foodborne outbreaks. Surveillance records with more granular temporal resolution and expanded geographic catchment areas can help improve the accuracy and precision of synchronization estimates for creating foodborne infection calendars, inspection schedules, and tracking multistate outbreaks. The public portal automatically compresses data during download, requiring individual year-by-year data extraction. Food recall reports show that a single outbreak is attributable to outbreaks in numerous states for these nine infections^44^. Failure to consider multistate outbreaks minimizes the utility of assessing cross-state synchronization of infection seasonality.

Given annual fiscal losses and food waste reported annually, our proposed synchronization metrics should be considered in order to mitigate seasonal co-infections, track multi-state outbreaks, and coordinate food inspection scheduling. Further investigation is needed to evaluate how synchronization metrics can identify common manmade drivers of infection during the packaging, processing, and transporting of food products. With thousands hospitalized or dying, millions of pounds of foodstuffs recalled, and billions of dollars lost annually, methods of describing and analyzing the seasonality and synchronization of foodborne infections can lead to important health benefits and cost savings for food producers, food retailers, and public health agencies alike.

## Data and methods

### Data sources

FoodNet reports confirmed cases from 650 randomly sampled clinical laboratories in select counties that reach roughly 15% of the US population (Supplementary Table S1) using both culture-dependent and culture-independent methods^8,55^. FoodNet Fast provides a publicly available subset of reports for confirmed annual infections, hospitalizations, and deaths as well as the monthly prevalence of confirmed infections. These data are available for seven bacterial infections (*Campylobacter*, *Listeria*, *Salmonella*, *Shigella*, Shiga toxin-producing *E.coli* (STEC), *Vibrio*, and *Yersinia*) and two protozoa (*Cryptosporidium* and *Cyclospora*) in ten select states: California (CA), Colorado (CO), Connecticut (CT), Georgia (GA), Maryland (MD), Minnesota (MN), New Mexico (NM), New York (NY), Oregon (OR), and Tennessee (TN) (Supplementary Fig. S1).

For each state, we downloaded all available annual infection profiles from 1996 to 2017. For each year, we created a monthly time series by multiplying the annual total of confirmed infections by the percentage of confirmed infections for each month of that year. National (US) estimates were generated by summing all ten states’ data. To draw comparisons between states, we calculated statewide population estimates by summing all mid-year (July 1st) populations of surveyed counties according to the year of their introduction into FoodNet (Supplementary Table S1). Annual county-level population estimates are made publicly available in the 1990, 2000, and 2010 US Census Bureau reports^56–58^. We calculated monthly rates per 1,000,000 persons by dividing monthly counts by population estimates and multiplying the product by 1,000,000. Results are presented as cases per 1,000,000 persons and abbreviated as ‘cpm.’

### Summary statistics for reported rates, trend, and seasonality analyses

To perform the synchronization analysis, we generated 99 individual monthly time series of reported rates (9 infections × 11 locations (each state and all states combined)). For each monthly time series of reported rates, we estimated summary statistics, including average rates and coefficients of L-skewness and L-kurtosis, which are superior on detecting spatiotemporal heterogeneity^59^. Estimates of L-skewness and L-kurtosis reflect the degree of departure of an empirical distribution from a symmetrical bell-shaped curve and the extent of extremes, respectively. Large values of L-skewness and L-kurtosis for a distribution of monthly rates are indicative of sporadic spikes, especially for infections with low overall rates. We used these estimates to identify infections with systematic periodic structures and infections with erratic temporal patterns. Infections with systematic periodic structures undergo trend, seasonality, and synchronization analyses based on annualized estimates of peak timing and amplitude. Infections with erratic temporal patterns were examined for trend and seasonality but only serial synchronization estimates were calculated.

To estimate average monthly rates from the compiled time series and adjust for left-skewed distributions, we applied a generalized linear model (GLM) with a negative binomial distribution and log-link function (Model 1). By exponentiating the model’s intercept, we calculated average monthly rates, exp{β_0_}, and their 95% confidence interval estimates, exp{β_0_ ± 1.96*se*}. This unadjusted model avoids biologically implausible, negative rates produced by the traditional arithmetic calculations (Supplementary Table S2).

Next, we developed four Negative Binomial Harmonic Regression (NBHR) models and applied these models for each infection in each state and all 10 states combined (Models 2–5). We explored the effects of linear, quadratic, and cubic trend terms, which were added in a stepwise manner to Model 2 containing solely harmonic seasonal oscillators.

1 $$\text{Model} \;1{:}\;\mathrm{ln}\left[\mathrm{E}\left({\mathrm{Y}}_{\mathrm{tds}}\right)\right]={\upbeta }_{0}$$

2 $$\text{Model} \;2{:}\;\mathrm{ln}\left[\mathrm{E}\left({\mathrm{Y}}_{\mathrm{tds}}\right)\right]={\upbeta }_{0}+ {\upbeta }_{\mathrm{s}}\left(\mathrm{sin}\left(2\mathrm{\pi \omega t}\right)\right)+{\upbeta }_{\mathrm{c}}\left(\mathrm{cos}\left(2\mathrm{\pi \omega t}\right)\right)$$

3 $$\text{Model} \;3{:}\;\mathrm{ln}\left[\mathrm{E}\left({\mathrm{Y}}_{\mathrm{tds}}\right)\right]={\upbeta }_{0}+ {\upbeta }_{\mathrm{s}}\left(\mathrm{sin}\left(2\mathrm{\pi \omega t}\right)\right)+{\upbeta }_{\mathrm{c}}\left(\mathrm{cos}\left(2\mathrm{\pi \omega t}\right)\right)+{\upbeta }_{1}\mathrm{t}$$

4 $$\text{Model} \;4{:}\;\mathrm{ln}\left[\mathrm{E}\left({\mathrm{Y}}_{\mathrm{tds}}\right)\right]={\upbeta }_{0}+ {\upbeta }_{\mathrm{s}}\left(\mathrm{sin}\left(2\mathrm{\pi \omega t}\right)\right)+{\upbeta }_{\mathrm{c}}\left(\mathrm{cos}\left(2\mathrm{\pi \omega t}\right)\right)+{\upbeta }_{1}\mathrm{t}+{\upbeta }_{2}{\mathrm{t}}^{2}$$

5 $$\text{Model}\; 5{:}\;\mathrm{ln}\left[\mathrm{E}\left({\mathrm{Y}}_{\mathrm{tds}}\right)\right]={\upbeta }_{0}+ {\upbeta }_{\mathrm{s}}\left(\mathrm{sin}\left(2\mathrm{\pi \omega t}\right)\right)+{\upbeta }_{\mathrm{c}}\left(\mathrm{cos}\left(2\mathrm{\pi \omega t}\right)\right)+{\upbeta }_{1}\mathrm{t}+{\upbeta }_{2}{\mathrm{t}}^{2}+{\upbeta }_{3}{\mathrm{t}}^{3}$$

where Y_tds_—time series of monthly rates of d-infection for t-month in s-state or all states combined; sin($2\mathrm{\pi \omega t}$) and cos($2\mathrm{\pi \omega t}$) periodic terms define seasonal oscillations with a frequency of ω = 1/M, where M = 12 to represent the length of the annual cycle in months; linear, quadratic, and cubic trend terms are defined by the consecutive month of the study from 1 to L and corresponding regression coefficients. The length of individual time series, L, varied by state according to its introduction into FoodNet: NM was the shortest with 168 months (beginning in 2004) and CA, CT, GA, MN, and OR were the longest being 264 months (beginning in 1996) (Supplementary Table S1).

We estimated peak timing, amplitude, and confidence intervals using the δ-methods (Supplementary Table S3) derived by MacNeill and Naumova^27^ with further modifications by Alarcon-Falconi, et al.^21^ for each infection in each state and all 10 states combined for the full duration of the study. Confidence intervals for peak timing and amplitude estimates are derived under the assumption of seasonal periodicity. As not all infections had consistent seasonal patterns, peak timing and confidence intervals can reach implausible values. Implausible peak timing estimates (values < 1 or > 13) occur when estimate variance exceeds 6 months for any infection or when peak timing estimates align with the beginning or end of the year (e.g. *Yersinia*). Implausible amplitude estimates (> 20) occur for erratic outbreaks or when estimate variance exceeds the average amplitude estimate (e.g. *Cyclospora*). Peak timing estimates are expressed in continuous month values from 1.0 (beginning of January) to 12.9(9) (end of December) according to the Gregorian calendar. Amplitude estimates are the midpoint of relative intensity reflecting the ratio between the disease rate at the peak (maximum rate) and the disease rate at the midpoint (median rate). Independent sample t-tests were used to determine statistically significant differences of peak timing estimates between states for the same infection and across infections within the same state.

Model goodness-of-fit was evaluated using the Akaike’s Information Criterion (AIC), Bayesian Information Criterion (BIC), and Root Mean Squared Error (RMSE). In addition, we assessed the contribution of each trend component by examining regression coefficients for linear, quadratic, and cubic trend terms. Depending on the sign, the linear term indicates overall increases (β_1_ > 0) or decreases (β_1_ < 0) while the quadratic and cubic terms indicate acceleration (β_2_ > 0) or deceleration (β_3_ > 0). We calculated the contribution of each term by multiplying each coefficient by the trend-associated time unit to recover the corresponding predicted rates. The contribution of each trend component (TC) to the overall fit was estimated as follows:

6 $${\text{TC}}_{{\text{k}}} = \beta_{k} t^{k} /(|\beta_{1} t| + |\beta_{2} t^{2} | + \, |\beta_{3} t^{3} |)*100\% ,$$

where TC_k_ is the contribution of k-component (k = 1, linear; k = 2, quadratic; and k = 3, cubic).

We explored the variability of seasonality estimates with respect to trend specification. We evaluated percentage differences of peak timing and amplitude estimates of Models 3–5 compared to Model 2 as follows:

7 $$\Delta {\text{P}}_{{\text{m}}} = \left( {{\text{P}}_{{\text{m}}} {-}{\text{ P}}_{{1}} } \right)/({\text{P}}_{{1}} )*{1}00\% \; {\text{and}} \; \Delta{\text{A}}_{{\text{m}}} = \left( {{\text{A}}_{{\text{m}}} {-}{\text{ A}}_{{1}} } \right)/({\text{A}}_{{1}} )*{1}00\% ,$$

where ΔP is percent difference of peak timing, P_m_, and amplitude, A_m_, estimates of *m*-model (m = 3,4,5) as compared to Model 2 (m = 2).

### Synchronization analyses

First, we calculated autocorrelations applied to monthly time series for the full duration of the study using Spearman correlations at 0-, 1-, 2-, and 3-month lags for each infection to confirm the strength of trend and seasonality components. For diseases with marked seasonality and overall trend, the strong serial synchronization reflected the similarities in temporal patterns. In some instances, prolonged periods of low incidence and occasional spikes could drive strong serial synchronization and thus be biased. For infections exhibiting well-marked irregularities, such as *Cyclospora* and *Yersinia*, serial synchronization metrics were likely to be biased, as evidenced by high values of L-skewness and L-kurtosis coefficients.

We then estimated three metrics of synchronization and compared them across infections within the same state and for each infection across states. Serial synchronization captures whether two infections or two locations share a similar temporal pattern. Phase-phase synchronization derives associations between peak timing estimates and identifies co-occurrences of outbreak timing and seasonal processes between infections or locations. Phase-amplitude synchronization examines seasonal behaviors of disease-state pairs and examines how the intensity of a seasonal peak of a foodborne illness varies in relation to its annual peak timing.

In order to conduct phase-phase and phase-amplitude synchronization analyses, we used annualized NBHR model (Model 1) estimates of seasonality characteristics: peak timing, amplitude, and their confidence intervals, using equations shown in Supplementary Table S3. Phase-phase and phase-amplitude synchronization metrics were calculated using Spearman correlations for 7 infections (excluding *Yersinia* and *Cyclospora*) in 11 locations (10 states and US national estimate) across a maximum of 22 years (varying by state). In total, we calculated 77 peak-amplitude pairs spanning a total of 1536 reporting years. Positive correlations for phase-phase synchronization indicate concordance of peak timing estimates between infections or states, e.g. if one infection or state peaks earlier in the calendar year, the other does also. Negative phase-phase synchronization correlations indicate that if one infection or state peaks earlier in the year, the other tends to peak later. Positive phase-amplitude synchronization indicates the magnitude of incidence increases when an infection peaks later in the year. Negative phase-amplitude synchronization indicates the magnitude of incidence decreases when an infection peaks later in the year. Determination of the association significance is based on the standard test for Spearman correlation at α < 0.05.

All statistical analyses were conducted using STATA (SE 15.1) software. All visualizations were designed and created using R Version 3.6.2 and Tableau Desktop 2019.1 software.

## Supplementary information

Supplementary Information.
