# Supplementary material for: Seasonal synchronization of foodborne outbreaks in the United States, 1996–2017
Source: Sci Rep. 2020 Oct 15;10:17500. doi: 10.1038/s41598-020-74435-9 (PMC7562704; doi:10.1038/s41598-020-74435-9)
Supplement: Supplementary file 1 — Supplementary Information. [file 41598_2020_74435_MOESM1_ESM.docx]

Supplementary Material

Seasonal synchronization of foodborne outbreaks in the United States, 1996-2017

Ryan B. Simpson ^1^, Bingjie Zhou ^1^, and Elena N. Naumova ^1,^*

^1^ Tufts University Friedman School of Nutrition Science and Policy; [Ryan.Simpson@tufts.edu](mailto:Ryan.Simpson@tufts.edu); <mailto:Bingjie.Zhou@tufts.edu>; [Elena.Naumova@tufts.edu](mailto:Elena.Naumova@tufts.edu)

***** Correspondence: [Elena.Naumova@tufts.edu](mailto:Elena.Naumova@tufts.edu)

**Supplementary Table S1.** An overview of state sampling area population (in millions) for the Foodborne Disease Surveillance Network (FoodNet) from 1996-2017.

| **State** |  |  |  |  | **Year** |  |  |  |  |
| --- | --- | --- | --- | --- | --- | --- | --- | --- | --- |
|  | 1996 | 1997 | 1998 | 1999 | 2000 | 2001 | 2002 | 2003 | 2004-2017* |
| **California (CA)** | 1.89 | 1.92 | 1.94 | 1.96 | 3.18 | 3.22 | 3.21 | 3.21 | 3.21 – 3.69 |
| **Colorado (CO)** |  |  |  |  |  | 2.10 | 2.46 | 2.51 | 2.53 – 3.15 |
| **Connecticut (CT)** | 1.62 | 1.62 | 3.27 | 3.28 | 3.40 | 3.41 | 3.43 | 3.46 | 3.49 – 3.59 |
| **Georgia (GA)** | 2.72 | 3.66 | 3.78 | 7.78 | 8.52 | 8.62 | 8.77 | 9.07 | 9.15 – 10.43 |
| **Maryland (MD)** |  |  | 2.44 | 2.45 | 2.52 | 4.24 | 5.43 | 5.50 | 5.55 – 6.05 |
| **Minnesota (MN)** | 4.65 | 4.69 | 4.73 | 4.78 | 4.93 | 4.98 | 5.02 | 5.05 | 5.09 – 5.58 |
| **New Mexico (NM)** |  |  |  |  |  |  |  |  | 1.90 – 2.09 |
| **New York (Y)** |  |  | 1.11 | 2.08 | 2.12 | 2.12 | 3.34 | 3.98 | 4.15 – 4.34 |
| **Oregon (OR)** | 3.20 | 3.24 | 3.28 | 3.32 | 3.43 | 3.47 | 3.51 | 3.55 | 3.57 – 4.14 |
| **Tennessee (TN)** |  |  |  |  | 2.88 | 2.91 | 2.95 | 5.84 | 5.91 – 6.72 |
| **All States (US)** | 14.08 | 15.13 | 20.55 | 25.65 | 30.98 | 35.07 | 38.12 | 42.17 | 44.55 – 49.78 |

**Supplementary Table S2.** Basic statistical characteristics: arithmetic monthly average rates per 1,000,000 persons accompanied by 95% confidence intervals, median rates (with lower and upper quartile intervals), standard skewness and kurtosis for the nine FoodNet reported infections for the United States and ten states from 1996-2017.

|  | **US** | **CA** | **CO** | **CT** | **GA** | **MD** | **MN** | **NM** | **NY** | **OR** | **TN** |
| --- | --- | --- | --- | --- | --- | --- | --- | --- | --- | --- | --- |
| **Bacteria, > 10.0 cpm** | | | | | | | | | | | |
| *Salmonella* | | | | | | | | | | | |
| LCI | 2.734 | 3.365 | 2.363 | 0.998 | -2.430 | 1.186 | 2.462 | -0.597 | -0.884 | 2.219 | 0.525 |
| Rate | 12.68 | 13.23 | 9.746 | 11.59 | 17.78 | 12.81 | 11.31 | 13.79 | 9.827 | 8.562 | 12.11 |
| UCI | 22.62 | 23.09 | 17.13 | 22.18 | 37.98 | 24.43 | 20.17 | 28.18 | 20.54 | 14.91 | 23.69 |
| LQR | 8.687 | 9.935 | 7.208 | 7.513 | 9.075 | 8.095 | 7.876 | 8.350 | 6.224 | 6.304 | 7.308 |
| Med | 11.48 | 12.05 | 8.847 | 10.09 | 14.44 | 11.66 | 10.11 | 11.56 | 8.434 | 8.055 | 10.79 |
| UQR | 16.29 | 16.00 | 11.80 | 14.76 | 26.29 | 16.03 | 13.32 | 17.27 | 12.73 | 10.06 | 15.88 |
| Skw | 0.664 | 1.225 | 1.438 | 1.106 | 0.916 | 0.992 | 1.225 | 1.572 | 3.196 | 1.174 | 0.943 |
| Krt | 2.485 | 5.381 | 7.196 | 4.072 | 3.181 | 3.808 | 4.753 | 6.358 | 24.54 | 5.433 | 3.645 |
| *Campylobacter* | | | | | | | | | | | |
| LCI | 2.421 | 6.352 | 0.855 | 0.877 | 0.778 | 0.338 | 2.088 | 0.028 | 1.408 | 4.658 | 1.336 |
| Rate | 12.17 | 26.60 | 11.99 | 14.34 | 6.452 | 7.270 | 15.37 | 14.59 | 11.60 | 16.08 | 5.699 |
| UCI | 21.92 | 46.84 | 23.12 | 27.81 | 12.13 | 14.20 | 28.65 | 29.16 | 21.80 | 27.50 | 10.06 |
| LQR | 8.548 | 20.13 | 7.952 | 9.990 | 4.428 | 4.606 | 10.33 | 8.808 | 7.928 | 11.47 | 4.046 |
| Med | 10.58 | 24.89 | 11.11 | 12.03 | 5.714 | 6.687 | 13.20 | 13.03 | 10.29 | 15.08 | 5.194 |
| UQR | 15.27 | 31.67 | 14.32 | 17.35 | 7.551 | 9.240 | 19.78 | 19.07 | 14.40 | 19.36 | 6.948 |
| Skw | 1.515 | 1.325 | 1.128 | 2.373 | 1.436 | 0.861 | 1.100 | 0.827 | 1.126 | 0.859 | 0.661 |
| Krt | 5.867 | 5.865 | 4.129 | 14.37 | 5.234 | 3.572 | 4.224 | 3.217 | 4.335 | 3.378 | 2.938 |
| **Bacteria, < 5.0 cpm** | | | | | | | | | | | |
| *Shigella* | | | | | | | | | | | |
| LCI | 0.474 | -2.375 | -2.641 | -1.226 | -3.103 | -5.042 | -5.817 | -2.381 | -2.331 | -1.413 | -3.418 |
| Rate | 4.835 | 7.551 | 3.193 | 1.733 | 8.693 | 3.266 | 3.972 | 4.655 | 1.741 | 2.083 | 5.330 |
| UCI | 9.195 | 17.48 | 9.027 | 4.691 | 20.49 | 11.57 | 13.76 | 11.69 | 5.813 | 5.580 | 14.08 |
| LQR | 3.259 | 4.057 | 1.290 | 0.841 | 4.593 | 1.239 | 1.427 | 2.282 | 0.528 | 0.992 | 2.314 |
| Med | 4.320 | 6.073 | 2.359 | 1.331 | 7.293 | 2.062 | 2.129 | 3.720 | 1.197 | 1.576 | 3.473 |
| UQR | 5.912 | 9.401 | 3.820 | 2.167 | 10.62 | 3.361 | 4.149 | 5.861 | 1.946 | 2.769 | 7.226 |
| Skw | 1.369 | 1.648 | 2.391 | 2.696 | 1.859 | 3.905 | 3.428 | 1.760 | 2.726 | 2.637 | 1.622 |
| Krt | 5.617 | 6.067 | 10.65 | 12.81 | 7.620 | 20.85 | 18.38 | 6.896 | 11.57 | 13.82 | 5.360 |
| *STEC* | | | | | | | | | | | |
| LCI | -0.601 | -1.686 | -1.755 | -1.412 | -0.515 | -0.499 | -2.401 | -0.850 | -3.096 | -2.333 | -0.694 |
| Rate | 1.711 | 1.610 | 2.455 | 1.663 | 0.654 | 0.814 | 3.616 | 1.641 | 1.707 | 2.585 | 1.256 |
| UCI | 4.024 | 4.907 | 6.665 | 4.738 | 1.823 | 2.127 | 9.632 | 4.132 | 6.509 | 7.504 | 3.206 |
| LQR | 0.797 | 0.529 | 0.880 | 0.640 | 0.250 | 0.346 | 1.227 | 0.567 | 0.537 | 0.871 | 0.536 |
| Med | 1.440 | 1.183 | 1.850 | 1.336 | 0.527 | 0.711 | 2.591 | 1.425 | 1.196 | 1.980 | 0.995 |
| UQR | 2.357 | 2.166 | 3.506 | 2.174 | 0.924 | 1.200 | 5.244 | 2.395 | 2.141 | 3.679 | 1.683 |
| Skw | 1.163 | 2.275 | 2.037 | 2.946 | 3.034 | 1.163 | 1.473 | 1.235 | 8.879 | 2.946 | 1.202 |
| Krt | 4.287 | 10.89 | 10.51 | 16.98 | 24.30 | 4.892 | 5.946 | 4.994 | 111.2 | 21.23 | 4.145 |
| **Bacteria, < 0.5 cpm** | | | | | | | | | | | |
| *Listeria* | | | | | | | | | | | |
| LCI | -0.011 | -0.360 | -0.704 | -0.506 | -0.162 | -0.263 | -0.259 | -0.805 | -0.559 | -0.285 | -0.174 |
| Rate | 0.256 | 0.374 | 0.213 | 0.466 | 0.204 | 0.284 | 0.138 | 0.198 | 0.331 | 0.248 | 0.153 |
| UCI | 0.523 | 1.109 | 1.131 | 1.438 | 0.569 | 0.831 | 0.536 | 1.201 | 1.222 | 0.780 | 0.480 |
| LQR | 0.155 | 0.000 | 0.000 | 0.000 | 0.094 | 0.000 | 0.000 | 0.000 | 0.000 | 0.000 | 0.000 |
| Med | 0.227 | 0.302 | 0.000 | 0.306 | 0.192 | 0.199 | 0.000 | 0.000 | 0.232 | 0.263 | 0.152 |
| UQR | 0.321 | 0.566 | 0.345 | 0.609 | 0.297 | 0.392 | 0.198 | 0.480 | 0.462 | 0.297 | 0.305 |
| Skw | 1.250 | 1.568 | 6.165 | 1.541 | 1.355 | 1.194 | 1.681 | 6.698 | 4.009 | 1.357 | 1.078 |
| Krt | 5.164 | 6.606 | 56.16 | 5.836 | 5.679 | 4.385 | 5.663 | 63.07 | 32.03 | 5.253 | 3.945 |
| *Vibrio* | | | | | | | | | | | |
| LCI | -0.307 | -1.319 | -0.430 | -0.985 | -0.252 | -0.735 | -0.387 | -0.275 | -0.414 | -0.805 | -0.217 |
| Rate | 0.270 | 0.625 | 0.189 | 0.414 | 0.221 | 0.486 | 0.153 | 0.078 | 0.174 | 0.286 | 0.126 |
| UCI | 0.847 | 2.570 | 0.809 | 1.814 | 0.693 | 1.707 | 0.693 | 0.432 | 0.761 | 1.377 | 0.469 |
| LQR | 0.075 | 0.000 | 0.000 | 0.000 | 0.000 | 0.000 | 0.000 | 0.000 | 0.000 | 0.000 | 0.000 |
| Med | 0.159 | 0.298 | 0.000 | 0.249 | 0.188 | 0.277 | 0.000 | 0.000 | 0.000 | 0.000 | 0.000 |
| UQR | 0.359 | 0.811 | 0.333 | 0.535 | 0.328 | 0.713 | 0.199 | 0.000 | 0.242 | 0.287 | 0.167 |
| Skw | 1.862 | 2.645 | 1.953 | 2.872 | 3.429 | 1.645 | 2.798 | 1.857 | 2.785 | 3.326 | 1.772 |
| Krt | 6.635 | 11.21 | 6.847 | 12.79 | 28.02 | 5.643 | 12.02 | 4.458 | 14.25 | 18.27 | 6.921 |
| *Yersinia* | | | | | | | | | | | |
| LCI | -0.128 | -0.652 | -0.386 | -0.420 | -1.038 | -0.245 | -0.279 | -0.372 | -0.322 | -0.333 | -0.318 |
| Rate | 0.384 | 0.510 | 0.237 | 0.394 | 0.489 | 0.200 | 0.409 | 0.110 | 0.352 | 0.420 | 0.274 |
| UCI | 0.895 | 1.673 | 0.860 | 1.208 | 2.016 | 0.645 | 1.098 | 0.592 | 1.026 | 1.174 | 0.867 |
| LQR | 0.238 | 0.000 | 0.000 | 0.000 | 0.115 | 0.000 | 0.190 | 0.000 | 0.000 | 0.000 | 0.000 |
| Med | 0.316 | 0.317 | 0.000 | 0.293 | 0.295 | 0.173 | 0.368 | 0.000 | 0.240 | 0.288 | 0.180 |
| UQR | 0.437 | 0.625 | 0.383 | 0.567 | 0.495 | 0.349 | 0.576 | 0.000 | 0.482 | 0.601 | 0.347 |
| Skw | 2.716 | 2.349 | 1.350 | 1.502 | 4.301 | 1.415 | 1.413 | 2.437 | 1.095 | 1.089 | 2.773 |
| Krt | 12.60 | 11.17 | 4.639 | 6.184 | 24.91 | 5.630 | 5.387 | 9.577 | 4.262 | 4.762 | 16.59 |
| **Protozoa, < 5.0 cpm** | | | | | | | | | | | |
| *Cryptosporidium* | | | | | | | | | | | |
| LCI | -0.887 | -1.922 | -2.131 | -1.289 | -0.118 | -0.686 | -2.810 | -4.278 | -14.54 | -1.814 | -1.541 |
| Rate | 2.044 | 1.824 | 1.292 | 1.133 | 2.164 | 0.706 | 4.290 | 3.697 | 2.127 | 2.547 | 1.168 |
| UCI | 4.975 | 5.569 | 4.714 | 3.554 | 4.446 | 2.098 | 11.39 | 11.67 | 18.80 | 6.908 | 3.877 |
| LQR | 1.147 | 0.680 | 0.394 | 0.324 | 1.373 | 0.205 | 1.840 | 1.352 | 0.480 | 0.826 | 0.371 |
| Med | 1.715 | 1.187 | 0.802 | 0.834 | 1.949 | 0.531 | 3.262 | 2.674 | 1.121 | 1.788 | 0.813 |
| UQR | 2.364 | 2.019 | 1.685 | 1.444 | 2.713 | 0.874 | 5.171 | 4.679 | 2.079 | 3.639 | 1.430 |
| Skw | 3.522 | 2.065 | 6.273 | 2.930 | 1.411 | 2.395 | 2.077 | 4.399 | 14.137 | 1.169 | 3.565 |
| Krt | 25.04 | 6.888 | 61.86 | 16.57 | 5.867 | 11.11 | 8.790 | 35.27 | 211.2 | 3.821 | 20.47 |
| **Protozoa, < 0.5 cpm** | | | | | | | | | | | |
| *Cyclospora* | | | | | | | | | | | |
| LCI | -0.426 | -1.064 | -0.844 | -1.644 | -0.818 | -0.397 | -0.359 | -0.363 | -0.379 | -0.229 | -0.135 |
| Rate | 0.098 | 0.141 | 0.133 | 0.311 | 0.206 | 0.068 | 0.074 | 0.078 | 0.055 | 0.036 | 0.028 |
| UCI | 0.622 | 1.347 | 1.110 | 2.265 | 1.229 | 0.532 | 0.506 | 0.519 | 0.488 | 0.302 | 0.191 |
| LQR | 0.000 | 0.000 | 0.000 | 0.000 | 0.000 | 0.000 | 0.000 | 0.000 | 0.000 | 0.000 | 0.000 |
| Med | 0.023 | 0.000 | 0.000 | 0.000 | 0.000 | 0.000 | 0.000 | 0.000 | 0.000 | 0.000 | 0.000 |
| UQR | 0.075 | 0.000 | 0.000 | 0.282 | 0.190 | 0.000 | 0.000 | 0.000 | 0.000 | 0.000 | 0.000 |
| Skw | 5.594 | 8.524 | 5.925 | 8.777 | 4.064 | 6.721 | 4.610 | 3.472 | 6.859 | 5.700 | 3.658 |
| Krt | 38.24 | 83.64 | 41.19 | 103.9 | 21.34 | 56.24 | 27.80 | 16.99 | 58.93 | 42.49 | 18.95 |

**Supplementary Table S3.** The δ-method equations for calculating peak timing and amplitude equations for any single-peaking seasonal infection using trend-adjusted Negative Binomial Harmonic Regression Models.

| Seasonality Feature | Model Equations | Equation Number |
| --- | --- | --- |
| Shift ($\emptyset$) | $\tan\emptyset=\frac{\sin\emptyset}{\cos\emptyset}$, thus: $\emptyset=arctan\left( \frac{\beta_{s}}{\beta_{c}} \right)$  $\mathrm{Var}\left( \emptyset\right)= \frac{{\beta_{c}}^{2}{\sigma_{s}}^{2}+ {\beta_{s}}^{2}{\sigma_{c}}^{2}- {2\sigma}_{\beta_{s}\beta_{c}}\beta_{s}\beta_{c}}{\left( {\beta_{c}}^{2}+ {\beta_{s}}^{2} \right)^{2}}$ | 1  2 |
| Peak Timing  (P_T_) | if $\beta_{s}>0 \& \beta_{c} >0$, then: $P_{T}= \left( \emptyset\right)\left( \frac{M}{2\pi} \right)$  if $\beta_{c}<0$, then: $P_{T}= \left( \emptyset+\pi\right)\left( \frac{M}{2\pi} \right)$  if $\beta_{s}<0 \& \beta_{c} >0$, then: $P_{T}= \left( \emptyset+2\pi\right)\left( \frac{M}{2\pi} \right)$  $95\% CI \left( P_{T} \right)= P_{T} \pm1.96\sqrt{Var(\emptyset)}\left( \frac{M}{2\pi} \right)$ | 3  4  5  6 |
| Amplitude  ($\gamma$) | $\gamma= e^{\vartheta}$, where $\vartheta=\sqrt{{\beta_{c}}^{2}+ {\beta_{s}}^{2}}$  $\mathrm{Var}\left( \vartheta\right)= \frac{{\beta_{c}}^{2}{\sigma_{c}}^{2}+ {\beta_{s}}^{2}{\sigma_{s}}^{2}+ {2\sigma}_{\beta_{s}\beta_{c}}\beta_{s}\beta_{c}}{{\beta_{c}}^{2}+ {\beta_{s}}^{2}}$  $95\% CI \left( \gamma\right)= e^{\vartheta\pm1.96\sqrt{\mathrm{Var}\left( \vartheta\right)}}$ | 7  8  9 |

**Supplementary Table S4.** Values of Root Mean Square Error (RMSE) for Models 1, 2, and 5 and the percentage differences between RMSE of Model 1 and 2 (Δ_12_) and Models 2 and 5 (Δ_25_).

|  | **US** | **CA** | **CO** | **CT** | **GA** | **MD** | **MN** | **NM** | **NY** | **OR** | **TN** |
| --- | --- | --- | --- | --- | --- | --- | --- | --- | --- | --- | --- |
| **Bacteria, > 10.0 cpm** | | | | | | | | | | | |
| *Salmonella* | | | | | | | | | | | |
| RMSE_1_ | 0.506 | 0.502 | 0.376 | 0.539 | 1.029 | 0.592 | 0.451 | 0.732 | 0.545 | 0.323 | 0.589 |
| RMSE_2_ | 0.183 | 0.352 | 0.304 | 0.366 | 0.541 | 0.307 | 0.303 | 0.502 | 0.376 | 0.260 | 0.315 |
| RMSE_5_ | 0.172 | 0.331 | 0.291 | 0.319 | 0.380 | 0.301 | 0.281 | 0.490 | 0.352 | 0.257 | 0.304 |
| Δ_12_ | 63.79 | 29.98 | 19.11 | 32.11 | 47.39 | 48.19 | 32.73 | 31.45 | 31.08 | 19.67 | 46.57 |
| Δ_25_ | 6.196 | 5.900 | 4.156 | 12.97 | 29.74 | 1.697 | 7.430 | 2.420 | 6.322 | 0.996 | 3.340 |
| *Campylobacter* | | | | | | | | | | | |
| RMSE_1_ | 0.497 | 1.031 | 0.567 | 0.686 | 0.289 | 0.353 | 0.676 | 0.741 | 0.519 | 0.582 | 0.222 |
| RMSE_2_ | 0.350 | 0.962 | 0.397 | 0.503 | 0.230 | 0.250 | 0.368 | 0.466 | 0.331 | 0.390 | 0.142 |
| RMSE_5_ | 0.182 | 0.484 | 0.337 | 0.465 | 0.157 | 0.200 | 0.345 | 0.432 | 0.269 | 0.332 | 0.138 |
| Δ_12_ | 29.42 | 6.677 | 30.02 | 26.69 | 20.27 | 29.04 | 45.52 | 37.11 | 36.15 | 32.88 | 36.08 |
| Δ_25_ | 48.00 | 49.68 | 15.07 | 7.505 | 31.65 | 20.14 | 6.473 | 7.187 | 18.97 | 14.94 | 2.513 |
| **Bacteria, < 5.0 cpm** | | | | | | | | | | | |
| *Shigella* | | | | | | | | | | | |
| RMSE_1_ | 0.222 | 0.505 | 0.297 | 0.151 | 0.601 | 0.423 | 0.498 | 0.358 | 0.207 | 0.178 | 0.445 |
| RMSE_2_ | 0.203 | 0.481 | 0.257 | 0.147 | 0.587 | 0.411 | 0.490 | 0.319 | 0.204 | 0.172 | 0.442 |
| RMSE_5_ | 0.172 | 0.360 | 0.204 | 0.111 | 0.556 | 0.367 | 0.470 | 0.294 | 0.201 | 0.148 | 0.435 |
| Δ_12_ | 8.473 | 4.895 | 13.30 | 2.319 | 2.236 | 2.801 | 1.685 | 10.93 | 1.493 | 3.323 | 0.778 |
| Δ_25_ | 15.35 | 25.14 | 20.75 | 24.45 | 5.257 | 10.70 | 4.154 | 7.743 | 1.410 | 14.30 | 1.639 |
| *STEC* | | | | | | | | | | | |
| RMSE_1_ | 0.118 | 0.168 | 0.214 | 0.157 | 0.060 | 0.067 | 0.306 | 0.127 | 0.245 | 0.250 | 0.099 |
| RMSE_2_ | 0.067 | 0.152 | 0.181 | 0.137 | 0.052 | 0.054 | 0.189 | 0.100 | 0.222 | 0.189 | 0.083 |
| RMSE_5_ | 0.056 | 0.103 | 0.171 | 0.134 | 0.048 | 0.052 | 0.179 | 0.094 | 0.218 | 0.183 | 0.069 |
| Δ_12_ | 43.17 | 9.614 | 15.66 | 12.76 | 11.91 | 19.96 | 38.29 | 21.31 | 9.059 | 24.51 | 16.28 |
| Δ_25_ | 15.98 | 32.24 | 5.327 | 1.863 | 8.230 | 3.106 | 5.295 | 5.461 | 1.950 | 3.369 | 17.12 |
| **Bacteria, < 0.5 cpm** | | | | | | | | | | | |
| *Listeria* | | | | | | | | | | | |
| RMSE_1_ | 0.014 | 0.037 | 0.047 | 0.050 | 0.019 | 0.028 | 0.020 | 0.051 | 0.045 | 0.027 | 0.017 |
| RMSE_2_ | 0.011 | 0.037 | 0.045 | 0.044 | 0.018 | 0.026 | 0.019 | 0.049 | 0.042 | 0.025 | 0.016 |
| RMSE_5_ | 0.009 | 0.037 | 0.044 | 0.043 | 0.017 | 0.025 | 0.018 | 0.048 | 0.041 | 0.025 | 0.016 |
| Δ_12_ | 20.36 | 0.300 | 4.041 | 11.55 | 1.512 | 8.296 | 5.372 | 3.127 | 7.101 | 7.292 | 1.229 |
| Δ_25_ | 13.53 | 1.169 | 1.645 | 2.860 | 7.333 | 1.698 | 4.321 | 1.899 | 2.981 | 1.577 | 0.361 |
| *Vibrio* | | | | | | | | | | | |
| RMSE_1_ | 0.029 | 0.099 | 0.032 | 0.071 | 0.024 | 0.062 | 0.027 | 0.018 | 0.030 | 0.056 | 0.017 |
| RMSE_2_ | 0.017 | 0.074 | 0.027 | 0.056 | 0.021 | 0.038 | 0.025 | 0.017 | 0.025 | 0.042 | 0.016 |
| RMSE_5_ | 0.013 | 0.072 | 0.027 | 0.046 | 0.021 | 0.029 | 0.020 | 0.017 | 0.023 | 0.038 | 0.015 |
| Δ_12_ | 42.90 | 25.62 | 13.63 | 21.27 | 12.83 | 38.11 | 8.060 | 2.732 | 16.01 | 24.56 | 10.89 |
| Δ_25_ | 19.68 | 2.770 | 0.815 | 17.29 | 0.847 | 23.74 | 19.43 | -0.057 | 6.606 | 8.422 | 1.488 |
| *Yersinia* | | | | | | | | | | | |
| RMSE_1_ | 0.026 | 0.059 | 0.032 | 0.041 | 0.078 | 0.023 | 0.035 | 0.024 | 0.034 | 0.038 | 0.030 |
| RMSE_2_ | 0.025 | 0.059 | 0.031 | 0.041 | 0.071 | 0.023 | 0.034 | 0.024 | 0.034 | 0.038 | 0.029 |
| RMSE_5_ | 0.018 | 0.050 | 0.030 | 0.038 | 0.050 | 0.021 | 0.029 | 0.023 | 0.034 | 0.036 | 0.028 |
| Δ_12_ | 3.361 | 0.091 | 1.171 | 0.154 | 8.768 | 0.336 | 1.721 | 1.137 | 0.409 | 0.949 | 5.262 |
| Δ_25_ | 28.96 | 15.24 | 3.528 | 8.607 | 30.21 | 6.373 | 16.98 | 3.030 | 0.468 | 6.348 | 3.380 |
| **Protozoa, < 5.0 cpm** | | | | | | | | | | | |
| *Cryptosporidium* | | | | | | | | | | | |
| RMSE_1_ | 0.149 | 0.191 | 0.174 | 0.123 | 0.116 | 0.071 | 0.362 | 0.406 | 0.849 | 0.222 | 0.138 |
| RMSE_2_ | 0.119 | 0.190 | 0.161 | 0.106 | 0.102 | 0.066 | 0.270 | 0.360 | 0.826 | 0.215 | 0.127 |
| RMSE_5_ | 0.109 | 0.107 | 0.151 | 0.103 | 0.087 | 0.048 | 0.239 | 0.279 | 0.813 | 0.157 | 0.106 |
| Δ_12_ | 20.37 | 0.453 | 7.295 | 13.72 | 12.63 | 7.526 | 25.18 | 11.18 | 2.727 | 3.165 | 8.178 |
| Δ_25_ | 8.00 | 43.49 | 6.430 | 2.852 | 14.72 | 26.76 | 11.67 | 22.46 | 1.559 | 26.98 | 15.98 |
| **Protozoa, < 0.5 cpm** | | | | | | | | | | | |
| *Cyclospora* | | | | | | | | | | | |
| RMSE_1_ | 0.027 | 0.061 | 0.050 | 0.100 | 0.052 | 0.024 | 0.022 | 0.022 | 0.022 | 0.013 | 0.008 |
| RMSE_2_ | 0.025 | 0.059 | 0.044 | 0.090 | 0.051 | 0.023 | 0.020 | 0.020 | 0.021 | 0.013 | 0.008 |
| RMSE_5_ | 0.019 | 0.050 | 0.022 | 0.080 | 0.034 | 0.014 | 0.013 | 0.019 | 0.019 | 0.013 | 0.007 |
| Δ_12_ | 7.061 | 4.124 | 11.36 | 9.693 | 2.312 | 4.281 | 9.957 | 9.349 | 6.190 | 0.498 | 7.656 |
| Δ_25_ | 24.93 | 15.07 | 49.91 | 11.20 | 33.01 | 37.77 | 35.91 | 7.559 | 7.251 | 1.684 | 6.695 |

**Supplementary Table S5.** Trend analyses: contribution of linear, quadratic, and cubic trend components from Model 5 for nine FoodNet reported infections for the United States and ten states from 1996-2017.

|  | **US** | **CA** | **CO** | **CT** | **GA** | **MD** | **MN** | **NM** | **NY** | **OR** | **TN** |
| --- | --- | --- | --- | --- | --- | --- | --- | --- | --- | --- | --- |
| **Bacteria, > 10.0 cpm** | | | | | | | | | | | |
| *Salmonella* | | | | | | | | | | | |
| Linear | -0.142 | -0.227 | 0.289 | -0.272 | 0.565 | -0.280 | -0.351 | 0.487 | -0.327 | -0.257 | -0.309 |
| Quadratic | 0.494 | 0.479 | -0.487 | 0.471 | -0.118 | 0.476 | 0.563 | -0.404 | 0.464 | 0.494 | 0.471 |
| Cubic | -0.364 | -0.294 | 0.224 | -0.257 | -0.317 | -0.245 | -0.086 | 0.109 | -0.209 | -0.249 | -0.220 |
| *Campylobacter* | | | | | | | | | | | |
| Linear | -0.260 | -0.227 | 0.363 | -0.251 | -0.266 | -0.275 | -0.476 | 0.396 | -0.292 | -0.199 | 0.333 |
| Quadratic | 0.474 | 0.470 | -0.468 | 0.481 | 0.467 | 0.491 | -0.098 | -0.447 | 0.478 | 0.485 | -0.471 |
| Cubic | -0.265 | -0.303 | 0.169 | -0.268 | -0.267 | -0.233 | 0.427 | 0.156 | -0.230 | -0.317 | 0.196 |
| **Bacteria, < 5.0 cpm** | | | | | | | | | | | |
| *Shigella* | | | | | | | | | | | |
| Linear | -0.878 | -0.364 | 0.250 | -0.292 | -0.203 | 0.244 | 0.157 | 0.405 | 0.150 | -0.313 | -0.259 |
| Quadratic | 0.120 | -0.224 | -0.502 | 0.468 | 0.463 | -0.488 | -0.492 | -0.451 | -0.507 | -0.258 | 0.477 |
| Cubic | -0.002 | 0.412 | 0.248 | -0.241 | -0.334 | 0.268 | 0.351 | 0.144 | 0.343 | 0.429 | -0.264 |
| STEC | | | | | | | | | | | |
| Linear | -0.303 | -0.235 | 0.785 | 0.094 | -0.010 | -0.164 | -0.304 | 0.468 | -0.386 | -0.105 | -0.313 |
| Quadratic | 0.500 | 0.041 | -0.061 | -0.482 | 0.608 | 0.515 | 0.506 | -0.413 | 0.462 | -0.219 | 0.492 |
| Cubic | -0.197 | 0.724 | -0.154 | 0.424 | -0.382 | -0.321 | -0.190 | 0.119 | -0.152 | 0.676 | -0.195 |
| **Bacteria, < 0.5 cpm** | | | | | | | | | | | |
| *Listeria* | | | | | | | | | | | |
| Linear | -0.382 | -0.250 | -0.214 | -0.281 | -0.608 | 0.167 | -0.534 | -0.355 | -0.351 | -0.264 | 0.298 |
| Quadratic | 0.454 | 0.479 | 0.506 | 0.471 | 0.257 | -0.502 | 0.334 | 0.466 | 0.453 | 0.488 | -0.482 |
| Cubic | -0.163 | -0.271 | -0.280 | -0.247 | 0.135 | 0.330 | 0.132 | -0.179 | -0.196 | -0.248 | 0.220 |
| *Vibrio* | | | | | | | | | | | |
| Linear | -0.119 | -0.197 | 0.341 | 0.485 | 0.232 | 0.578 | -0.165 | -0.206 | 0.421 | -0.166 | 0.266 |
| Quadratic | 0.554 | 0.489 | -0.460 | 0.182 | -0.474 | -0.318 | 0.530 | 0.557 | -0.417 | 0.510 | 0.411 |
| Cubic | -0.327 | -0.315 | 0.199 | -0.333 | 0.294 | 0.104 | -0.305 | -0.236 | 0.162 | -0.324 | -0.323 |
| *Yersinia* | | | | | | | | | | | |
| Linear | -0.420 | -0.274 | -0.564 | -0.247 | -0.269 | -0.321 | -0.031 | 0.442 | -0.281 | -0.417 | 0.308 |
| Quadratic | 0.456 | -0.316 | -0.049 | 0.469 | 0.460 | 0.469 | -0.440 | -0.427 | 0.501 | 0.484 | -0.503 |
| Cubic | -0.124 | 0.410 | 0.387 | -0.284 | -0.271 | -0.210 | 0.529 | 0.131 | -0.218 | 0.099 | 0.190 |
| **Protozoa, < 5.0 cpm** | | | | | | | | | | | |
| *Cryptosporidium* | | | | | | | | | | | |
| Linear | -0.251 | -0.352 | 0.369 | -0.249 | -0.217 | -0.236 | -0.209 | 0.467 | 0.294 | -0.142 | 0.372 |
| Quadratic | 0.497 | 0.461 | -0.452 | 0.491 | 0.519 | 0.520 | 0.518 | -0.415 | -0.476 | 0.536 | -0.402 |
| Cubic | -0.252 | -0.188 | 0.179 | -0.260 | -0.264 | -0.244 | -0.273 | 0.119 | 0.230 | -0.322 | 0.226 |
| **Protozoa, < 0.5 cpm** | | | | | | | | | | | |
| *Cyclospora* | | | | | | | | | | | |
| Linear | -0.381 | -0.556 | 0.603 | -0.335 | -0.352 | 0.261 | 0.385 | -0.721 | 0.317 | 0.506 | 0.388 |
| Quadratic | 0.475 | 0.374 | -0.338 | 0.482 | 0.465 | -0.485 | -0.469 | 0.260 | -0.497 | -0.406 | -0.452 |
| Cubic | -0.144 | -0.071 | 0.059 | -0.183 | -0.182 | 0.254 | 0.146 | -0.019 | 0.186 | 0.088 | 0.160 |

**Supplementary Table S6.** Percentage differences of peak timing and amplitude estimates for Models 3, 4, and 5 compared to Model 2.

|  | **US** | **CA** | **CO** | **CT** | **GA** | **MD** | **MN** | **NM** | **NY** | **OR** | **TN** |  |
| --- | --- | --- | --- | --- | --- | --- | --- | --- | --- | --- | --- | --- |
| **Peak Timing** | | | | | | | | | | | |  |
| **Bacteria, > 10.0 cpm** | | | | | | | | | | | |  |
| *Salmonella* | | | | | | | | | | | |  |
| Model 3 | -0.042 | 0.113 | 0.306 | 0.254 | -0.120 | 0.055 | -0.197 | -0.114 | 0.156 | -0.060 | 0.031 |  |
| Model 4 | -0.040 | 0.116 | 0.314 | 0.278 | -0.097 | 0.056 | -0.215 | -0.102 | 0.167 | -0.064 | 0.031 |  |
| Model 5 | 0.021 | 0.362 | 0.055 | 0.491 | -0.075 | 0.191 | -0.202 | -0.203 | 0.288 | 0.048 | 0.163 |  |
| *Campylobacter* | | | | | | | | | | | |  |
| Model 3 | 0.436 | 1.324 | 0.445 | 0.114 | 0.655 | -0.422 | 0.116 | -0.014 | 0.039 | -0.020 | 0.098 |  |
| Model 4 | 0.501 | 1.420 | 0.406 | 0.124 | 0.737 | -0.480 | 0.121 | -0.025 | 0.039 | -0.021 | 0.095 | |
| Model 5 | 0.976 | 2.666 | 0.216 | 0.418 | 1.205 | -0.087 | 0.094 | -0.282 | 0.461 | 0.564 | -0.073 | |
| **Bacteria, < 5.0 cpm** | | | | | | | | | | | | |
| *Shigella* | | | | | | | | | | | | |
| Model 3 | 0.431 | 0.762 | 0.330 | 1.506 | 0.294 | 0.707 | 0.520 | 0.296 | 0.267 | 0.274 | 0.035 | |
| Model 4 | 0.434 | 0.876 | 0.383 | 1.942 | 0.289 | 0.650 | 0.566 | 0.297 | 0.281 | 0.295 | 0.078 | |
| Model 5 | 0.434 | 0.730 | 0.143 | 2.800 | 0.492 | -0.727 | -0.618 | 0.208 | -0.014 | 0.234 | 0.087 | |
| STEC | | | | | | | | | | | | |
| Model 3 | -0.088 | -0.651 | -0.521 | 0.012 | -0.579 | -0.276 | -0.073 | -0.315 | 0.080 | -0.179 | -0.741 | |
| Model 4 | -0.116 | -0.928 | -0.461 | 0.011 | -0.580 | -0.245 | -0.088 | -0.275 | 0.094 | -0.215 | -0.815 | |
| Model 5 | -0.017 | -1.053 | -0.440 | -0.231 | -0.493 | -0.104 | -0.042 | -0.635 | 0.168 | -0.261 | -0.571 | |
| **Bacteria, < 0.5 cpm** | | | | | | | | | | | | |
| *Listeria* | | | | | | | | | | | | |
| Model 3 | 0.258 | 0.852 | -0.069 | 0.152 | 1.934 | 0.179 | 0.061 | -0.018 | 0.161 | 0.008 | 0.206 | |
| Model 4 | 0.287 | 0.938 | -0.045 | 0.169 | 2.306 | 0.196 | 0.053 | -0.001 | 0.175 | -0.001 | 0.206 | |
| Model 5 | 0.345 | 2.510 | 0.035 | 0.288 | 2.181 | -0.050 | 0.041 | 0.100 | 0.240 | 0.197 | -0.024 | |
| *Vibrio* | | | | | | | | | | | | |
| Model 3 | -0.170 | -0.028 | -0.113 | -0.267 | -0.130 | -0.208 | -0.692 | -0.061 | -0.322 | -0.127 | -0.252 | |
| Model 4 | -0.174 | -0.029 | -0.121 | -0.219 | -0.125 | -0.198 | -0.732 | -0.045 | -0.296 | -0.126 | -0.234 | |
| Model 5 | -0.134 | 0.069 | -0.248 | -0.198 | -0.305 | -0.204 | -0.498 | -0.027 | -0.349 | -0.048 | -0.208 | |
| *Yersinia* | | | | | | | | | | | | |
| Model 3 | -12.22 | -41.39 | -0.954 | 3.851 | -10.46 | -2.993 | -0.445 | -0.410 | -0.543 | 0.057 | -3.477 | |
| Model 4 | -16.22 | -13.28 | -1.383 | 4.379 | -13.45 | -3.406 | -0.737 | -0.730 | -0.531 | 0.305 | -3.223 | |
| Model 5 | -18.57 | -41.49 | -1.672 | 7.010 | -19.31 | -7.982 | -2.547 | -2.800 | -0.300 | 0.313 | -2.562 | |
| **Protozoa, < 5.0 cpm** | | | | | | | | | | | | |
| *Cryptosporidium* | | | | | | | | | | | | |
| Model 3 | -0.251 | 2.026 | -0.449 | -0.163 | -0.525 | -0.737 | -0.315 | -0.114 | -0.010 | -1.822 | -0.677 | |
| Model 4 | -0.296 | 2.594 | -0.435 | -0.201 | -0.514 | -0.818 | -0.338 | -0.054 | -0.002 | -1.683 | -0.757 | |
| Model 5 | -0.062 | 3.109 | -0.976 | 0.131 | -0.344 | -0.590 | -0.212 | -0.909 | -0.187 | -1.142 | -0.856 | |
| **Protozoa, < 0.5 cpm** | | | | | | | | | | | | |
| *Cyclospora* | | | | | | | | | | | | |
| Model 3 | 0.216 | 0.295 | -0.320 | 0.012 | 1.357 | -0.690 | -0.266 | -0.030 | -0.071 | 0.173 | 0.041 | |
| Model 4 | 0.487 | 1.166 | -1.192 | 0.021 | 2.819 | -1.190 | -0.668 | 0.112 | -0.030 | 0.316 | 0.020 | |
| Model 5 | 0.771 | 1.647 | -1.710 | 0.166 | 3.993 | -2.117 | -1.569 | 0.183 | -0.319 | -1.479 | -0.322 | |

|  | **US** | **CA** | **CO** | **CT** | **GA** | **MD** | **MN** | **NM** | **NY** | **OR** | **TN** |
| --- | --- | --- | --- | --- | --- | --- | --- | --- | --- | --- | --- |
| **Amplitude** | | | | | | | | | | | |
| **Bacteria, > 10.0 cpm** | | | | | | | | | | | |
| *Salmonella* | | | | | | | | | | | |
| Model 3 | -0.061 | 0.096 | 0.215 | 0.225 | -0.356 | 0.072 | -0.115 | -0.182 | 0.119 | -0.020 | 0.053 |
| Model 4 | -0.064 | 0.108 | 0.238 | 0.273 | -0.322 | 0.082 | -0.108 | -0.198 | 0.148 | -0.012 | 0.050 |
| Model 5 | 0.027 | 0.329 | 0.054 | 0.482 | -0.259 | 0.266 | -0.101 | -0.352 | 0.248 | 0.026 | 0.285 |
| *Campylobacter* | | | | | | | | | | | |
| Model 3 | 0.280 | 0.612 | 0.311 | 0.066 | 0.224 | -0.279 | 0.105 | -0.021 | 0.037 | -0.010 | 0.053 |
| Model 4 | 0.368 | 0.683 | 0.254 | 0.098 | 0.285 | -0.279 | 0.123 | 0.041 | 0.085 | -0.006 | 0.040 |
| Model 5 | 0.728 | 1.445 | 0.116 | 0.286 | 0.489 | -0.021 | 0.097 | -0.345 | 0.527 | 0.308 | -0.050 |
| **Bacteria, < 5.0 cpm** | | | | | | | | | | | |
| *Shigella* | | | | | | | | | | | |
| Model 3 | 0.913 | 1.550 | 1.959 | 1.087 | 0.845 | 0.698 | 0.520 | 1.653 | 0.696 | 1.771 | 0.579 |
| Model 4 | 0.925 | 1.906 | 2.546 | 1.578 | 0.808 | 0.618 | 0.599 | 1.683 | 0.833 | 2.201 | 0.486 |
| Model 5 | 0.926 | 1.562 | 1.020 | 2.441 | 1.527 | -0.534 | -0.483 | 1.128 | 0.062 | 1.722 | 1.176 |
| STEC | | | | | | | | | | | |
| Model 3 | -0.183 | -1.721 | -0.564 | 0.013 | -0.440 | -0.593 | -0.177 | -0.449 | 0.220 | -0.543 | -0.618 |
| Model 4 | -0.163 | -2.230 | -0.534 | 0.055 | -0.442 | -0.553 | -0.166 | -0.444 | 0.355 | -0.597 | -0.641 |
| Model 5 | 0.043 | -2.495 | -0.515 | -0.194 | -0.383 | -0.256 | -0.055 | -0.865 | 0.568 | -0.725 | -0.466 |
| **Bacteria, < 0.5 cpm** | | | | | | | | | | | |
| *Listeria* | | | | | | | | | | | |
| Model 3 | 0.627 | 0.103 | -0.364 | 0.434 | 0.043 | 0.507 | 0.892 | -0.113 | 0.759 | 0.022 | 0.398 |
| Model 4 | 0.761 | 0.145 | -0.328 | 0.530 | 0.114 | 0.622 | 1.365 | -0.170 | 0.889 | 0.078 | 0.395 |
| Model 5 | 0.915 | 0.392 | 0.106 | 0.902 | 0.104 | -0.068 | 1.164 | 0.511 | 1.230 | 0.666 | -0.046 |
| *Vibrio* | | | | | | | | | | | |
| Model 3 | -0.420 | -0.091 | -0.287 | -0.732 | -0.226 | -0.408 | -1.248 | -0.308 | -0.682 | -0.365 | -0.363 |
| Model 4 | -0.425 | -0.089 | -0.284 | -0.633 | -0.225 | -0.399 | -1.300 | -0.271 | -0.648 | -0.363 | -0.356 |
| Model 5 | -0.333 | 0.253 | -0.567 | -0.583 | -0.509 | -0.409 | -0.946 | -0.184 | -0.733 | -0.148 | -0.322 |
| *Yersinia* | | | | | | | | | | | |
| Model 3 | -0.496 | 0.608 | -0.745 | 0.863 | -0.878 | -1.093 | -0.130 | -1.291 | 0.358 | 1.137 | -0.833 |
| Model 4 | -0.742 | 0.809 | -0.830 | 1.073 | -1.203 | -1.510 | -0.016 | -1.581 | 0.381 | 1.467 | -0.746 |
| Model 5 | -0.824 | 0.570 | -1.016 | 2.106 | -1.751 | -2.908 | -0.403 | -5.630 | 0.224 | 1.549 | -0.585 |
| **Protozoa, < 5.0 cpm** | | | | | | | | | | | |
| *Cryptosporidium* | | | | | | | | | | | |
| Model 3 | -0.620 | 2.997 | -1.315 | -0.501 | -1.036 | -2.242 | -0.761 | -0.275 | -0.018 | -2.090 | -2.229 |
| Model 4 | -0.675 | 4.951 | -1.288 | -0.544 | -1.022 | -2.423 | -0.794 | -0.256 | -0.818 | -1.970 | -2.416 |
| Model 5 | -0.100 | 6.617 | -2.489 | 0.532 | -0.707 | -1.821 | -0.507 | -1.674 | -2.370 | -1.439 | -2.675 |
| **Protozoa, < 0.5 cpm** | | | | | | | | | | | |
| *Cyclospora* | | | | | | | | | | | |
| Model 3 | -0.013 | -0.207 | 0.023 | -0.002 | 1.926 | -0.639 | -0.099 | 0.020 | -0.024 | 0.101 | 0.003 |
| Model 4 | 0.737 | 0.164 | 2.420 | 0.268 | 5.789 | -0.568 | 0.131 | 0.859 | 0.327 | 0.165 | 0.119 |
| Model 5 | 0.840 | 0.060 | 3.266 | 0.287 | 9.116 | -0.073 | 1.668 | 0.798 | 0.435 | -0.355 | 0.589 |

Supplementary Table S7. Autocorrelations for lags 1-3 for nine FoodNet reported infections for the United States and ten states from 1996-2017. Bold correlation coefficients are significant (α ≥ 0.05).

|  | **US** | **CA** | **CO** | **CT** | **GA** | **MD** | **MN** | **NM** | **NY** | **OR** | **TN** |
| --- | --- | --- | --- | --- | --- | --- | --- | --- | --- | --- | --- |
| **Bacteria, > 10.0 cpm** | | | | | | | | | | | |
| *Salmonella* | | | | | | | | | | | |
| Lag 1 | **0.807** | **0.596** | **0.468** | **0.647** | **0.816** | **0.704** | **0.621** | **0.625** | **0.665** | **0.532** | **0.731** |
| Lag 2 | **0.455** | **0.299** | **0.219** | **0.381** | **0.486** | **0.396** | **0.371** | **0.377** | **0.354** | **0.271** | **0.407** |
| Lag 3 | 0.014 | 0.027 | 0.070 | 0.063 | 0.069 | 0.011 | 0.056 | 0.025 | 0.015 | 0.009 | -0.001 |
| *Campylobacter* | | | | | | | | | | | |
| Lag 1 | **0.807** | **0.745** | **0.602** | **0.628** | **0.705** | **0.719** | **0.697** | **0.669** | **0.673** | **0.634** | **0.592** |
| Lag 2 | **0.538** | **0.649** | **0.367** | **0.399** | **0.480** | **0.438** | **0.393** | **0.383** | **0.402** | **0.393** | **0.256** |
| Lag 3 | **0.190** | **0.539** | 0.069 | 0.066 | **0.216** | **0.143** | 0.038 | -0.017 | 0.101 | 0.048 | -0.053 |
| **Bacteria, < 5.0 cpm** | | | | | | | | | | | |
| *Shigella* | | | | | | | | | | | |
| Lag 1 | **0.842** | **0.732** | **0.588** | **0.443** | **0.843** | **0.652** | **0.763** | **0.598** | **0.471** | **0.460** | **0.773** |
| Lag 2 | **0.680** | **0.581** | **0.451** | **0.277** | **0.726** | **0.522** | **0.678** | **0.362** | **0.352** | **0.396** | **0.667** |
| Lag 3 | **0.506** | **0.454** | **0.284** | **0.292** | **0.631** | **0.379** | **0.586** | **0.174** | **0.297** | **0.247** | **0.572** |
| STEC | | | | | | | | | | | |
| Lag 1 | **0.803** | **0.576** | **0.535** | **0.346** | **0.592** | **0.527** | **0.674** | **0.338** | **0.450** | **0.636** | **0.563** |
| Lag 2 | **0.497** | **0.425** | **0.396** | **0.170** | **0.472** | **0.371** | **0.373** | **0.323** | **0.226** | **0.396** | **0.385** |
| Lag 3 | **0.126** | **0.310** | **0.306** | 0.004 | **0.293** | **0.146** | 0.047 | 0.073 | 0.083 | **0.169** | **0.194** |
| **Bacteria, < 0.5 cpm** | | | | | | | | | | | |
| *Listeria* | | | | | | | | | | | |
| Lag 1 | **0.553** | **0.112** | 0.022 | **0.217** | **0.250** | **0.145** | **0.152** | 0.122 | **0.165** | 0.111 | 0.069 |
| Lag 2 | **0.365** | 0.006 | 0.020 | **0.102** | **0.239** | 0.087 | **0.133** | -0.026 | 0.072 | **0.124** | 0.067 |
| Lag 3 | 0.060 | 0.006 | 0.024 | -0.017 | **0.131** | 0.032 | 0.099 | -0.059 | -0.027 | -0.052 | 0.030 |
| *Vibrio* | | | | | | | | | | | |
| Lag 1 | **0.699** | **0.461** | **0.163** | **0.399** | **0.318** | **0.628** | **0.166** | 0.014 | **0.310** | **0.432** | **0.207** |
| Lag 2 | **0.416** | **0.244** | 0.085 | **0.260** | **0.229** | **0.366** | **0.158** | -0.155 | **0.159** | **0.133** | 0.081 |
| Lag 3 | 0.065 | 0.000 | **0.142** | 0.055 | 0.068 | 0.049 | 0.110 | -0.076 | -0.034 | -0.046 | 0.041 |
| *Yersinia* | | | | | | | | | | | |
| Lag 1 | **0.453** | **0.240** | 0.000 | **0.128** | **0.363** | **0.174** | **0.348** | 0.056 | -0.014 | 0.092 | **0.173** |
| Lag 2 | **0.275** | **0.228** | 0.092 | 0.102 | **0.276** | 0.089 | **0.272** | 0.075 | -0.005 | **0.148** | -0.070 |
| Lag 3 | **0.239** | **0.165** | 0.019 | **0.232** | **0.156** | **0.168** | **0.220** | -0.105 | -0.067 | -0.028 | 0.019 |
| **Protozoa, < 5.0 cpm** | | | | | | | | | | | |
| *Cryptosporidium* | | | | | | | | | | | |
| Lag 1 | **0.784** | **0.446** | **0.439** | **0.489** | **0.610** | **0.504** | **0.700** | **0.692** | **0.484** | **0.755** | **0.584** |
| Lag 2 | **0.508** | **0.375** | **0.371** | **0.243** | **0.446** | **0.369** | **0.427** | **0.562** | **0.265** | **0.702** | **0.414** |
| Lag 3 | **0.264** | **0.429** | **0.199** | 0.059 | **0.248** | **0.341** | **0.193** | **0.397** | 0.121 | **0.658** | **0.287** |
| **Protozoa, < 0.5 cpm** | | | | | | | | | | | |
| *Cyclospora* | | | | | | | | | | | |
| Lag 1 | **0.461** | -0.090 | **0.273** | **0.384** | **0.514** | **0.162** | **0.259** | 0.087 | 0.071 | -0.124 | -0.001 |
| Lag 2 | **0.227** | 0.020 | -0.098 | 0.096 | **0.274** | 0.041 | 0.065 | -0.064 | -0.031 | 0.021 | 0.125 |
| Lag 3 | 0.004 | 0.012 | -0.003 | -0.085 | **0.166** | 0.058 | 0.040 | -0.067 | -0.096 | 0.015 | -0.100 |

**Supplementary Table S8.** Serial synchronization between nine FoodNet reported infections for the United States from 1996-2017. Analyses are conducted between the first infection at lag 0 and the second infection at lags 0, 1, 2, and 3 as well in the reverse order. **Bold** correlation coefficients are significant (α ≥ 0.05).

| **Comparison** | **-3** | **-2** | **-1** | **0** | **1** | **2** | **3** |
| --- | --- | --- | --- | --- | --- | --- | --- |
| Salmonella-Campylobacter | **-0.144** | **0.244** | **0.585** | **0.780** | **0.727** | **0.501** | **0.134** |
| Salmonella-Shigella | **0.135** | **0.300** | **0.390** | **0.392** | **0.257** | 0.050 | **-0.171** |
| Salmonella-STEC | 0.010 | **0.404** | **0.701** | **0.806** | **0.683** | **0.383** | 0.007 |
| Salmonella-Listeria | **0.135** | **0.398** | **0.556** | **0.582** | **0.408** | **0.155** | **-0.165** |
| Salmonella-Vibrio | -0.039 | **0.359** | **0.673** | **0.818** | **0.713** | **0.435** | 0.052 |
| Salmonella-Yersinia | 0.004 | -0.087 | **-0.138** | **-0.145** | **-0.157** | **-0.135** | -0.041 |
| Salmonella-Cryptosporidium | **0.162** | **0.416** | **0.578** | **0.594** | **0.425** | **0.178** | -0.119 |
| Salmonella-Cyclospora | **-0.353** | -0.098 | **0.184** | **0.438** | **0.583** | **0.582** | **0.381** |
| Campylobacter -Shigella | **0.315** | **0.432** | **0.476** | **0.441** | **0.322** | **0.141** | -0.049 |
| Campylobacter -STEC | 0.119 | **0.432** | **0.630** | **0.661** | **0.493** | **0.192** | **-0.126** |
| Campylobacter -Listeria | **0.360** | **0.603** | **0.689** | **0.632** | **0.421** | **0.182** | -0.064 |
| Campylobacter -Vibrio | 0.012 | **0.354** | **0.581** | **0.626** | **0.459** | **0.181** | **-0.150** |
| Campylobacter -Yersinia | **0.180** | **0.138** | 0.099 | 0.119 | **0.140** | 0.110 | **0.205** |
| Campylobacter -Cryptosporidium | **0.254** | **0.481** | **0.553** | **0.487** | **0.283** | 0.043 | **-0.166** |
| Campylobacter -Cyclospora | **-0.258** | -0.012 | **0.281** | **0.509** | **0.552** | **0.465** | **0.262** |
| Shigella -STEC | **-0.216** | -0.006 | **0.158** | **0.263** | **0.273** | **0.187** | 0.017 |
| Shigella -Listeria | 0.037 | **0.153** | **0.277** | **0.310** | **0.297** | **0.230** | 0.089 |
| Shigella -Vibrio | **-0.291** | -0.069 | 0.120 | **0.246** | **0.247** | **0.189** | 0.033 |
| Shigella -Yersinia | **0.146** | **0.132** | 0.079 | 0.013 | -0.057 | -0.022 | 0.045 |
| Shigella -Cryptosporidium | **-0.214** | -0.101 | -0.006 | 0.051 | 0.034 | -0.049 | **-0.194** |
| Shigella -Cyclospora | **-0.276** | **-0.154** | -0.035 | 0.093 | **0.194** | **0.265** | **0.273** |
| STEC -Listeria | 0.088 | **0.356** | **0.520** | **0.507** | **0.362** | 0.107 | **-0.149** |
| STEC -Vibrio | 0.004 | **0.373** | **0.657** | **0.784** | **0.710** | **0.446** | 0.100 |
| STEC -Yersinia | 0.023 | -0.066 | **-0.139** | -0.121 | -0.104 | -0.098 | -0.049 |
| STEC -Cryptosporidium | **0.296** | **0.569** | **0.748** | **0.750** | **0.569** | **0.302** | 0.038 |
| STEC -Cyclospora | **-0.289** | -0.112 | 0.116 | **0.357** | **0.506** | **0.544** | **0.379** |
| Listeria -Vibrio | **-0.255** | 0.048 | **0.328** | **0.487** | **0.484** | **0.325** | 0.056 |
| Listeria -Yersinia | **0.313** | **0.134** | 0.082 | 0.119 | **0.191** | **0.225** | **0.235** |
| Listeria -Cryptosporidium | -0.062 | **0.173** | **0.404** | **0.474** | **0.373** | **0.180** | -0.011 |
| Listeria -Cyclospora | **-0.331** | -0.122 | 0.084 | **0.284** | **0.348** | **0.435** | **0.274** |
| Vibrio -Yersinia | -0.090 | **-0.141** | **-0.194** | **-0.193** | **-0.177** | **-0.128** | -0.077 |
| Vibrio -Cryptosporidium | **0.229** | **0.504** | **0.681** | **0.647** | **0.466** | **0.181** | -0.087 |
| Vibrio -Cyclospora | **-0.314** | -0.096 | **0.168** | **0.404** | **0.529** | **0.478** | **0.329** |
| Yersinia -Cryptosporidium | -0.016 | 0.001 | -0.031 | -0.067 | **-0.159** | -0.067 | 0.109 |
| Yersinia -Cyclospora | 0.091 | 0.050 | -0.022 | -0.001 | -0.001 | 0.015 | -0.081 |
| Cryptosporidium -Cyclospora | **-0.245** | **-0.186** | -0.026 | **0.181** | **0.345** | **0.456** | **0.380** |

**Supplementary Table S9.** Phase-phase synchronization Spearman correlations across time series at lags 0 calculated on seven FoodNet reported infections for the United States and ten states from 1996-2017. **Bold** correlation coefficients indicate significance (α < 0.05).

| Comparison | US | CA | CO | CT | GA | MD | MN | NM | NY | OR | TN |
| --- | --- | --- | --- | --- | --- | --- | --- | --- | --- | --- | --- |
| Salmonella-Campylobacter | 0.072 | **-0.499** | -0.034 | -0.045 | -0.066 | 0.158 | **0.600** | 0.051 | 0.005 | **0.443** | 0.150 |
| Salmonella-Shigella | 0.198 | 0.182 | -0.007 | -0.211 | 0.281 | 0.101 | 0.342 | 0.099 | -0.388 | -0.042 | 0.044 |
| Salmonella-STEC | 0.085 | 0.247 | 0.407 | 0.042 | 0.418 | 0.147 | 0.408 | **0.622** | 0.023 | 0.169 | 0.228 |
| Salmonella-Listeria | 0.163 | 0.361 | 0.118 | **0.581** | 0.077 | 0.084 | -0.004 | 0.169 | 0.400 | -0.187 | 0.092 |
| Salmonella-Vibrio | -0.199 | -0.117 | -0.145 | 0.021 | 0.264 | **0.558** | 0.001 | -0.170 | 0.146 | -0.098 | 0.214 |
| Salmonella-Cryptosporidium | **0.460** | -0.365 | 0.142 | 0.401 | 0.233 | -0.005 | 0.178 | -0.358 | 0.215 | -0.047 | -0.104 |
| Campylobacter-Shigella | 0.165 | -0.126 | 0.118 | 0.226 | 0.120 | 0.147 | **0.457** | 0.165 | 0.123 | -0.015 | **0.507** |
| Campylobacter-STEC | -0.294 | -0.243 | 0.252 | **0.532** | **-0.571** | -0.014 | **0.452** | 0.196 | -0.122 | -0.072 | 0.383 |
| Campylobacter-Listeria | 0.013 | 0.005 | 0.478 | 0.180 | 0.137 | **0.456** | 0.116 | 0.086 | -0.059 | 0.321 | 0.127 |
| Campylobacter-Vibrio | -0.076 | 0.031 | -0.025 | -0.116 | -0.244 | 0.132 | 0.091 | -0.549 | -0.061 | -0.380 | **0.488** |
| Campylobacter-Cryptosporidium | 0.149 | 0.390 | 0.228 | -0.084 | 0.370 | 0.289 | 0.065 | 0.090 | 0.063 | -0.256 | 0.451 |
| Shigella-STEC | 0.113 | -0.076 | -0.047 | 0.103 | -0.011 | -0.230 | 0.287 | -0.138 | -0.116 | 0.249 | **0.515** |
| Shigella-Listeria | 0.277 | -0.135 | -0.225 | -0.010 | 0.326 | -0.421 | 0.089 | -0.024 | -0.006 | -0.244 | 0.240 |
| Shigella-Vibrio | -0.132 | 0.164 | -0.059 | -0.126 | -0.037 | 0.251 | 0.203 | 0.055 | 0.059 | -0.033 | -0.075 |
| Shigella-Cryptosporidium | **0.508** | 0.022 | 0.103 | 0.010 | 0.186 | -0.014 | -0.030 | 0.116 | -0.047 | 0.249 | -0.075 |
| STEC-Listeria | 0.093 | **0.578** | 0.346 | 0.154 | -0.144 | 0.161 | -0.056 | -0.244 | 0.183 | 0.152 | 0.296 |
| STEC-Vibrio | -0.101 | -0.055 | -0.027 | -0.099 | 0.128 | -0.008 | -0.208 | -0.187 | 0.046 | 0.158 | 0.148 |
| STEC-Cryptosporidium | 0.079 | 0.084 | 0.284 | 0.056 | -0.218 | 0.051 | 0.231 | -0.420 | 0.068 | -0.131 | 0.209 |
| Listeria-Vibrio | 0.003 | -0.333 | -0.299 | 0.006 | 0.043 | -0.188 | -0.275 | 0.302 | -0.088 | 0.067 | -0.013 |
| Listeria-Cryptosporidium | **0.506** | 0.099 | -0.061 | 0.240 | 0.030 | 0.159 | 0.029 | -0.147 | -0.089 | -0.286 | 0.055 |
| Vibrio-Cryptosporidium | -0.062 | -0.030 | 0.130 | 0.251 | -0.272 | 0.205 | -0.157 | -0.093 | -0.079 | -0.235 | 0.282 |

**Supplementary Table S10.** Phase-amplitude synchronization estimated with Spearman correlation coefficients between the peak timing and amplitude estimates for disease-state dyads of 7 FoodNet reported infections in 10 states and the US from 1996-2017.

|  | US | CA | CO | CT | GA | MD | MN | NM | NY | OR | TN |
| --- | --- | --- | --- | --- | --- | --- | --- | --- | --- | --- | --- |
| Salmonella | 0.154 | 0.094 | -0.211 | 0.031 | -0.185 | 0.417 | -0.142 | 0.160 | 0.311 | 0.294 | 0.412 |
| Campylobacter | -0.169 | -0.193 | -0.233 | -0.216 | -0.128 | -0.275 | 0.181 | 0.108 | 0.144 | 0.064 | -0.346 |
| Shigella | 0.242 | **0.634** | 0.172 | 0.255 | **0.635** | 0.054 | **0.425** | 0.011 | 0.161 | -0.153 | 0.193 |
| STEC | **0.496** | -0.302 | -0.056 | -0.060 | **0.580** | -0.086 | 0.361 | -0.068 | 0.123 | 0.217 | 0.040 |
| Listeria | -0.360 | -0.072 | 0.260 | 0.150 | 0.165 | -0.131 | 0.344 | -0.138 | -0.008 | 0.239 | 0.278 |
| Vibrio | 0.051 | -0.276 | 0.120 | -0.053 | 0.032 | -0.110 | -0.015 | 0.462 | 0.284 | 0.174 | -0.049 |
| Cryptosporidium | 0.153 | -0.256 | -0.022 | **0.477** | 0.388 | 0.203 | 0.079 | 0.464 | 0.069 | 0.127 | 0.346 |

**Note:** significant correlation coefficients are shown in bold.

**
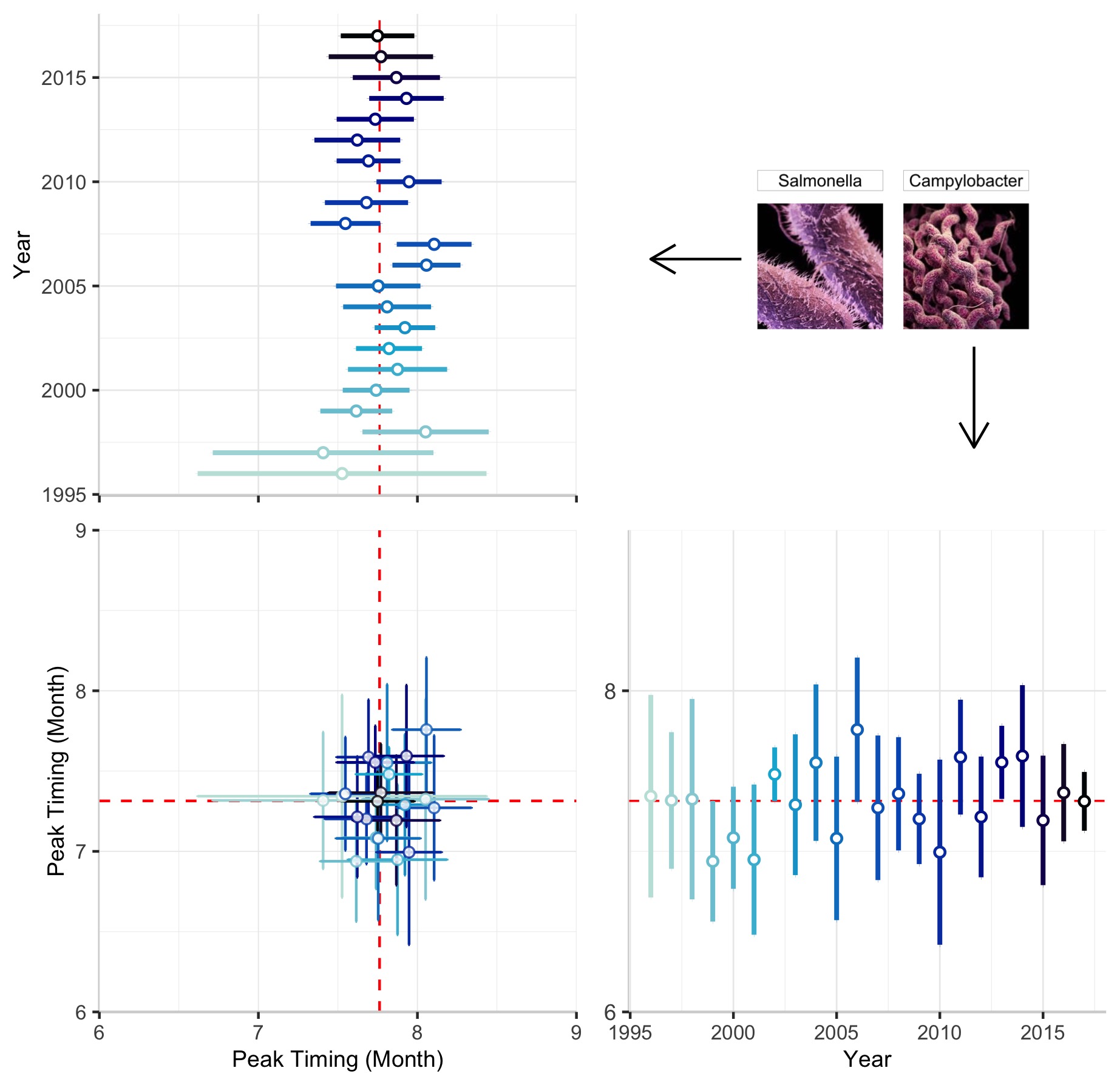
**

**Supplementary Figure S1.** The relationship of annual peak timing between *Shigella* and *Cryptosporidium* in the US from 1996-2017. Panels include two forest plots of infection peak timing by year and a scatter plot of peak timing between them. Dashed red lines indicate the average peak timing for each infection. The colour shade indicates the year of reporting (more historic vs. more recent data).

**
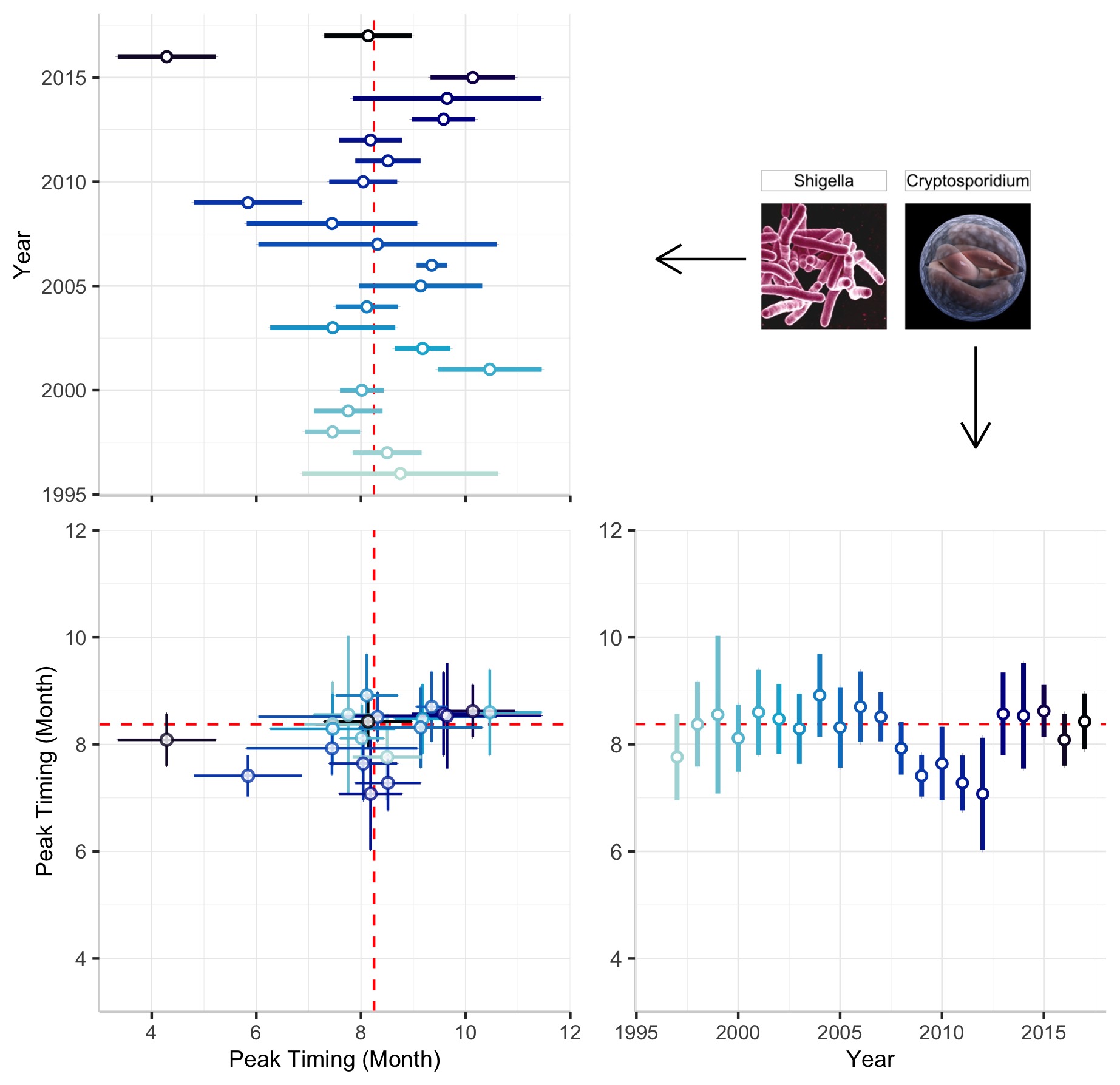
**

**Supplementary Figure S2.** The relationship of annual peak timing between *Salmonella* and *Campylobacter* in the US from 1996-2017. Panels include two forest plots of infection peak timing by year and a scatter plot of peak timing between them. Dashed red lines indicate the average peak timing for each infection. The colour shade indicates the year of reporting (more historic vs. more recent data).
